# From sport to everyday movement: a school-based “DON'T STAY STILL!” initiative to address sedentary behavior in children

**DOI:** 10.3389/fspor.2026.1979089

**Published:** 2026-10-14

**Authors:** Gianvincenzo Zuccotti, Franco Bruno Ascani, Assunta Ferdinanda Romeo, PODiaCar Team, Valeria Calcaterra

**Affiliations:** 1 Pediatric Department, Buzzi Children’s Hospital, Milan, Italy; 2 Department of Biomedical and Clinical Sciences, University of Milan, Milan, Italy; 3 International Federation of Sport Cinema and Television, Milan, Italy; 4 Project 101128946-PODiaCar-EU4H-2022-PJ-3, Buzzi Children’s Hospital, Milan, Italy; 5 Department of Internal Medicine and Therapeutics, University of Pavia, Pavia, Italy

**Keywords:** active break, children, physical activity, school, sedentary behavior

## Abstract

**Introduction:**

Sedentary behavior is a relevant health concern in childhood and adolescence and may remain high even among children who participate in structured physical activity. Schools therefore represent a key setting for reducing prolonged sitting by integrating brief opportunities for movement into everyday routines. “DON'T STAY STILL!” was developed as a school-based pathway specifically targeting sedentary behavior through short, feasible, and inclusive “moments of movement.”

**Methods:**

The initiative was implemented during the 2025–2026 school year within the 63rd edition of the “Trofei di Milano-Cortina 2026” programme. Teachers received the PODiaCar “DON'T STAY STILL!” educational guide and supporting videos and incorporated brief active breaks into routine school activities. Students were also involved through drawings and short videos focused on movement and active lifestyles. Programme participation, implementation, and teacher feedback were assessed descriptively; no objective pre/post measures of sedentary behavior or physical activity were collected.

**Results:**

The overall programme involved 118 schools and 61,053 students aged 6–13 years, 52% of whom were girls. Within this network, the “DON'T STAY STILL!” pathway generated 2,992 student outputs, including individual and class drawings and videos, while 3,090 students participated in guided “moments of movement” during school-based educational sessions using PODiaCar video exercises. Teacher feedback was positive, although acceptability was not assessed using a validated questionnaire.

**Conclusions:**

“DON'T STAY STILL!” provides a feasible school-based approach for addressing sedentary behavior by embedding brief movement opportunities into routine educational activities. Its implementation within a large and diverse school network, together with substantial student participation and positive teacher feedback, supports its potential as a scalable strategy for promoting more active school environments. The findings support feasibility, participation, and preliminary acceptability, but do not establish effectiveness in reducing sedentary behavior. Future prospective studies should assess its effects on sedentary time, physical activity, and related behavioral and health outcomes.

## 1 Introduction

Sedentary behavior has become a prominent feature of contemporary childhood (1–3). Time spent sitting during lessons, homework, transport, screen-based entertainment, and other low-movement activities may occupy a substantial proportion of the waking day. This pattern is associated with adverse indicators of adiposity, cardiometabolic health, fitness, psychosocial well-being, and sleep in children and adolescents (4–6). Prevention should therefore address not only participation in sport, but also how movement and sedentary time are distributed across the day (7–9).

Sedentary behavior and physical inactivity are related but distinct concepts (10, 11). A child may meet recommendations for moderate-to-vigorous physical activity while still accumulating prolonged periods of sitting (12). Conversely, interrupting sedentary time does not necessarily require formal exercise: standing, walking, stretching, and brief classroom-based movement can all contribute to a more active daily pattern (11). This distinction broadens the health-education message from “do more sport” to “move more often” (1, 11).

Schools are particularly well positioned to translate this concept into practice. Because prolonged sitting is embedded in many learning activities, the school day offers repeated opportunities to introduce brief movement, reinforce health literacy, engage peer groups, and involve teachers as facilitators (13, 14). School-based interventions can therefore complement physical education by integrating movement into ordinary lessons and transitions (13–16).

Active classroom breaks are a practical strategy for interrupting sedentary time (13–16). They require neither dedicated sports facilities nor major timetable changes and can be adapted to age, ability, available space, and curricular needs. In addition to increasing movement, classroom-based physical activity has been associated with favorable effects on attention, on-task behavior, enjoyment, and selected cognitive and academic outcomes (17, 18).

For health-promotion messages to be sustainable, they should also be simple, participatory, inclusive, and embedded in familiar educational contexts (19, 20). Combining movement with creativity, peer interaction, audiovisual communication, and multidisciplinary learning may help children translate an abstract health message into small, repeatable actions (21).

The “Trofei di Milano-Cortina” is a long-standing Italian school-based initiative integrating sport, education, culture, social participation, and Olympic and Paralympic values (21). Its 63rd edition, included in the Milano Cortina 2026 Gen26 Education Programme, was delivered during the 2025–2026 school year in primary and lower-secondary schools and incorporated educational pathways on physical activity, nutrition, balanced technology use, creativity, inclusion, and sport.

Within this framework, the “DON'T STAY STILL!” initiative focused specifically on sedentary behavior through the concept of the “moment of movement”: a short, feasible episode of activity introduced during lessons, between classes, or at other appropriate times. The approach complements physical education and organized sport by emphasizing the value of brief, repeated movement throughout the school day.

This report describes the rationale, structure, implementation, reach, and initial teacher acceptability of “DON'T STAY STILL!” and examines how a large school-based programme can extend the promotion of sport toward the prevention of sedentary behavior.

## 2 Methods

### 2.1 Programme context

The 63rd edition of “Trofei di Milano-Cortina 2026—Education, Culture and Sport for Young People” was organized as a year-long school project for children aged 6–13 years attending primary and lower-secondary schools. Included in the Milano Cortina 2026 Gen26 Education Programme, it combined sport and education within a broader health-promotion framework.

The programme was integrated with PODiaCar (Combating Pediatric Obesity: From a Predictive Tool for Type 2 Diabetes and Cardiovascular Risk to Health Education Programs; Project 101128946-PODiaCar-EU4H-2022-PJ-3), an EU-supported project coordinated by Buzzi Children's Hospital in Milan. PODiaCar contributed preventive expertise and structured health-education content, with a specific focus on sedentary behavior and everyday movement through the “DON'T STAY STILL!” pathway.

Activities included an educational phase conducted throughout the school year and a sporting phase culminating in final events. School registration closed on 16 December 2025, formative activities were conducted from January to April 2026, educational outputs were due by 21 April 2026, and the final sporting phase was scheduled for 6–8 May 2026 at Arena Brera in Milan. Participation was free of charge.

### 2.2 School recruitment

Schools were recruited through the established dissemination network of the “Trofei di Milano-Cortina” programme. Primary and lower-secondary schools in Lombardy and Veneto received institutional communications outlining the educational aims, planned activities, participation procedures, materials, and annual schedule.

The official regulations and participation form were subsequently distributed through educational and institutional networks. Participation was voluntary and free of charge. There was no predefined limit on the number of students per enrolled school. Teachers served as school-level facilitators, coordinating activities, distributing materials, implementing classroom tasks, and supporting student participation.

### 2.3 Main components of the programme

Although the present manuscript specifically focuses on sedentary behavior, “DON'T STAY STILL!” was conceived as one component of the broader multidimensional educational framework of the 63rd edition. The programme integrated educational, preventive, creative, and sporting activities designed to promote active lifestyles, healthy behaviors, inclusion, participation, and Olympic and Paralympic values. These activities were organized into seven interconnected areas (Table 1).

- “The European Sport Flag”: a creative activity centered on European identity, friendship, respect, and teamwork;
- “DON'T STAY STILL!”: the sedentary-behavior prevention pathway based on brief “moments of movement” integrated into the school day;
- “Education 4.0”: an educational pathway promoting balanced technology use and awareness of excessive screen time;
- “Healthy Nutrition—Prevention in Action”: a health-education pathway focused on healthy and balanced nutrition;
- “Your Games in a Video”: student-produced audiovisual content inspired by Olympic and Paralympic stories, athletes, values, and ceremonies;
- “Screenings—Sporting Emotions in the Classroom”: school-based or online screenings and discussions addressing sport, health, education, inclusion, and Olympic values;
- “Your Competitions”: age-adapted sporting activities and competitions promoting structured physical activity and participation.

**Table 1:** Main components of the 63rd edition and their contribution to the overall health-education model.

Area	Main activity	Contribution to the overall health-education model
The European Sport Flag	Creative design activity	Values, identity, friendship, respect, teamwork
DON’T STAY STILL!	Active breaks and student drawings/videos	Sedentary-behavior prevention and everyday movement
Education 4.0	Digital detox and balanced technology use	Reduction of excessive screen-related sedentary time
Healthy Nutrition—Prevention in Action	Healthy-menu educational activity	Nutrition literacy and healthy lifestyle
Your Games in a Video	Student audiovisual productions	Creativity, participation, Olympic/Paralympic values
Screenings—Sporting Emotions in the Classroom	School/online screenings and discussion	Health, inclusion, sport culture, educational engagement
Your Competitions	Age-adapted sporting activities	Structured physical activity, participation, and sport

Together, these components provided complementary opportunities to address everyday movement, structured physical activity, nutrition, technology use, creativity, inclusion, and shared values. Within this broader framework, “DON'T STAY STILL!” represented the specific pathway dedicated to reducing sedentary behavior and promoting the integration of movement into everyday school routines.

### 2.4 Educational materials and teacher support

Teachers received the PODiaCar educational guide “DON'T STAY STILL!” (Supplementary Material 1), which provided age-appropriate information on sedentary behavior, related physical and psychosocial consequences, physical activity, active classroom breaks, movement-integrated learning, family strategies, inclusion, and healthy screen use.

Example videos demonstrated practical “moments of movement,” with differentiated content for children and teachers to reflect their distinct roles and needs: child-focused videos promoted direct participation in age-appropriate movement activities, whereas teacher-focused videos provided practical guidance on how to introduce, adapt, and integrate these activities into classroom routines.

### 2.5 “moments of movement”

The core operational element was the “moment of movement”: a brief episode of motor activity introduced during lessons, class transitions, or other appropriate times to interrupt prolonged sitting without replacing curricular physical education or organized sport.

Examples included:

- simple stretching exercises performed beside the desk or in a circle;
- playful motor activities, imitation, balance tasks, and small jumps;
- brief rhythmic sequences using music, counting, hand claps, or foot movements;
- short walks inside the classroom, corridor, courtyard, or other available school spaces.

The guide recommended brief, frequent active breaks, generally lasting 5–10 min and adaptable to age, functional ability, available space, and classroom context. No fixed frequency was prescribed, and actual frequency and duration were not systematically recorded across classes. Representative illustrative examples are shown in Figure 1.

**Figure 1:**
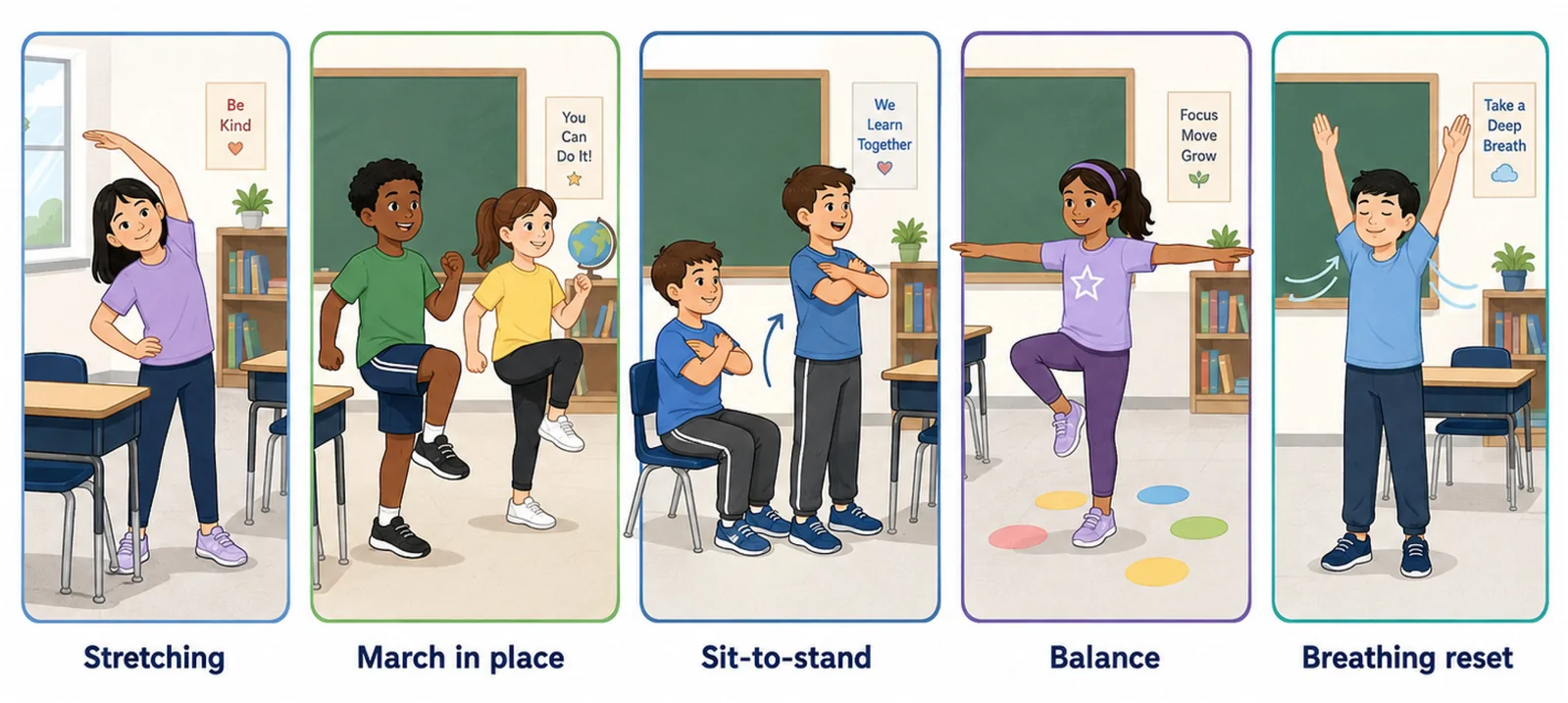
Illustrative representation of brief school-based active breaks in the classroom setting.

### 2.6 Multidisciplinary learning and creative participation

Movement was also integrated into curricular learning, for example through syllable- or rhyme-associated movement in language activities, miming verbs in foreign-language lessons, representing numbers or geometric shapes with the body in mathematics, and activities related to breathing, balance, or body movement in science.

Students were invited to create a drawing or short video showing their own “moment of movement” at school, thereby reinforcing health literacy and encouraging active communication of the preventive message.

### 2.7 Inclusion

The intervention was designed to be adaptable and inclusive. Teachers modified activities according to age, motor ability, space, and educational needs so that participation was not limited to children already involved in sport.

### 2.8 Data collection and descriptive analysis

Programme implementation was summarized using aggregate participation data provided by the organizing team, including the number of schools, students, student-generated outputs, and participation in guided “moments of movement”. Teacher acceptability was described from informal feedback collected during implementation; no validated questionnaire was administered. Categorical data were reported as counts and percentages; no inferential statistical tests were performed. No objective pre/post assessment of sedentary behavior or physical activity was performed.

## 3 Results

### 3.1 Programme participation and implementation

The 63rd edition of the “Trofei di Milano-Cortina 2026” programme involved 118 schools, across Lombardy and Veneto, with approximately two thirds located in Lombardy and one third in Veneto. Overall, 61,053 students aged 6–13 years participated, 52% of whom were girls. These data reflect the overall reach of the programme, whereas specific participation in “DON'T STAY STILL!” was documented through student-generated outputs and guided “moments of movement”; school-level implementation of classroom active breaks was not systematically tracked. Participation was also open to children attending the hospital school at Buzzi Children's Hospital in Milan**,** allowing hospitalized students to take part in the educational activities. The final sporting events attracted more than 20,000 spectators.

Within this broader programme, teachers implemented the “DON'T STAY STILL!” pathway using the PODiaCar educational guide and supporting videos and incorporated brief “moments of movement” into routine school activities. The activities did not require dedicated facilities or specific equipment and were therefore adaptable to heterogeneous primary- and lower-secondary-school settings.

### 3.2 Student participation and educational outputs

The “DON'T STAY STILL!” educational pathway generated 2,992 student outputs, including individual and class drawings and videos focused on movement and active lifestyles. These materials illustrated how students interpreted the importance of interrupting prolonged sitting and incorporated movement into the school day.

“Moments of movement” were also included in school-based screening and educational sessions, during which 3,090 students took part in brief active breaks by replicating exercises shown in the PODiaCar video guides.

The creative outputs also reflected links with curricular learning and other programme themes, including healthy nutrition, balanced technology use, inclusion, creativity, Olympic and Paralympic values, and sport. For privacy reasons, student-produced videos are not included as Supplementary Material.

### 3.3 Teacher acceptability

Teachers providing feedback expressed a positive opinion of “DON'T STAY STILL!”, reporting that the pathway was easy to integrate into routine school activities and relevant for promoting awareness of sedentary behavior and active lifestyles. The educational materials and example videos were also considered useful for supporting classroom implementation.

As no validated satisfaction or acceptability questionnaire was administered, these findings should be interpreted as descriptive evidence of perceived feasibility and educational value rather than standardized acceptability outcomes.

### 3.4 Stakeholder engagement

The programme was supported by a multisectoral network of educational, healthcare, sporting, and community stakeholders. Key contributors included the International Federation of Sport Cinema and Television (FICTS), the PODiaCar consortium, Buzzi Children's Hospital (Milano, Italy), participating schools and educational institutions, school staff, and families.

This broad involvement supported programme dissemination, school participation, health-education activities, and community engagement, strengthening the connection between schools, families, healthcare, sport, and the local community.

## 4 Discussion

The 63rd edition of the “Trofei di Milano-Cortina 2026” illustrates a broad school-based health-promotion model that integrates structured sport with educational and preventive activities.

Within this framework, “DON'T STAY STILL!” addresses a complementary aspect of active living by emphasizing that participation in physical education or organized sport does not necessarily prevent prolonged sedentary time during the rest of the day. Reducing and regularly interrupting sitting therefore represents an additional target for school-based health promotion (13–16).

This target is particularly relevant in schools, where sitting is inherent to many lessons. The objective is not to eliminate seated learning, but to avoid unnecessarily prolonged uninterrupted periods through simple opportunities to stand, stretch, walk, or perform brief motor tasks without substantially disrupting teaching (13–16). The low-resource nature of these activities, which require neither dedicated facilities nor specific equipment, may also facilitate their implementation and adaptation across different educational contexts**,** including settings in which students may have temporary or health-related limitations.

Embedding “DON'T STAY STILL!” within the broader programme may strengthen its educational impact. Other modules address healthy eating, balanced technology use, sport, inclusion, and creativity; in particular, “Education 4.0” targets excessive digital exposure, while “Your Competitions” provides structured opportunities for physical activity. Together, these components address complementary aspects of daily lifestyle, thereby contributing to a broader and more integrated approach to the promotion of healthy behaviours among children and adolescents.

The multidisciplinary approach may also favor sustainability by allowing teachers to link movement with language, mathematics, science, art, and other subjects (22–24). This integration may normalize movement within the learning process, while evidence from classroom-based interventions suggests that active breaks can increase physical activity and may support attention, on-task behavior, cognition, and selected academic outcomes (17, 18).

The creative component further engages students by asking them to interpret the health message, translate it into practical behavior, and communicate it through drawings or videos. This active reformulation of educational content is conceptually consistent with teach-back approaches, in which learners demonstrate understanding by restating or applying information in their own words or actions; such strategies have been associated with improved knowledge, recall, and self-management across different educational and healthcare settings (25). In the school context, opportunities to express understanding and receive positive reinforcement may also support motivation, engagement, and confidence in learning (26). Thus, student-generated outputs may contribute not only to creativity and peer communication, but also to active processing, ownership of the preventive message, and health literacy (25, 26). The production of 2,992 individual and class outputs indicates substantial engagement with the educational message, while the participation of 3,090 students in guided “moments of movement” during school-based educational sessions provides additional evidence of direct involvement in the practical component of the initiative.

Positive teacher feedback is encouraging because educator engagement is essential for implementation (26, 27). Nevertheless, the absence of a validated instrument limits interpretation, and future editions should include standardized measures of acceptability, feasibility, fidelity, and sustainability.

The scale of participation and community engagement demonstrates the capacity of the “Trofei di Milano-Cortina network to disseminate preventive messages broadly. By incorporating sedentary behavior and digital lifestyles, the 63rd edition extends an established sport-and-education model to contemporary health-promotion priorities (28, 29). The involvement of schools, families, educational staff, healthcare professionals, sports organizations, and institutional partners also highlights the value of a multisectoral approach in supporting dissemination and implementation (30).

Several limitations should be acknowledged. This report primarily describes programme implementation, reach, participation, and acceptability rather than clinical or behavioral effectiveness. No objective pre/post measures of sedentary time or physical activity were collected; therefore, the findings cannot demonstrate that the programme reduced sedentary behavior or increased physical activity. Although student engagement was documented through creative outputs and guided “moments of movement”**,** the frequency and duration of active breaks were not systematically recorded across schools, and teacher acceptability was assessed descriptively without a validated questionnaire. In addition, student-produced drawings and videos were not subjected to a standardized qualitative or quantitative analysis. Some of these limitations also reflect the organizational complexity of implementing a large school-based programme. Future evaluations should include process indicators, standardized teacher and student acceptability measures, and, when feasible, objective assessment of sedentary time and physical activity using accelerometry or wearable devices. A prospective design would help distinguish the contribution of “DON'T STAY STILL!” from that of the broader programme.

Despite these limitations, the initiative shows how a long-standing sport-and-education programme can evolve into a broader health-promotion platform while preserving the social and educational value of sport.

## 5 Conclusion

The “Trofei di Milano-Cortina 2026” provides a broad school-based framework for promoting active and healthy lifestyles.

Within this framework, “DON'T STAY STILL!” specifically targets sedentary behavior by incorporating brief, feasible “moments of movement” into everyday school activities. The documented participation and positive teacher feedback support the feasibility and preliminary acceptability of the approach, while effectiveness requires prospective evaluation with standardized measures.

Future prospective studies are needed to assess behavioral and health outcomes and to further evaluate the scalability of the approach across different school contexts.

## Data Availability

The raw data supporting the conclusions of this article will be made available by the authors, without undue reservation.

## Group members of PODiaCar Team

ITALY: Virginia Rossi (Buzzi Children’s Hospital and University of Milano, Milano, Italy), Elvira Verduci (Buzzi Children’s Hospital and University of Milano, Milano, Italy), Savina Mannarino (Buzzi Children’s Hospital and University of Milano, Milano, Italy), Giulia Fiore (Buzzi Children’s Hospital and University of Milano, Milano, Italy), Lucia Labati (Buzzi Children’s Hospital and University of Milano, Milano, Italy), Matteo Vandoni (University of Pavia, Italy), Alessandro Gatti (University of Pavia, Italy), Luca Marin (University of Pavia, Italy), Vittoria Carnevale Pellino (University of Pavia, Italy), Caterina Cavallo (University of Pavia, Italy), Agnese Pirazzi (University of Pavia, Italy); SPAIN: Cristina Campoy (University of Granada, Granada, Spain), Mireia Escudero Marin (University of Granada, Granada, Spain), Rocio Bonillo Leon (University of Granada, Granada, Spain), Mercedes García-Bermúdez (University of Granada, Granada, Spain), Inmaculada Guerrero (University of Granada, Granada, Spain); LUXEMBOURG: Camilo Corbellini (University of Applied Sciences, Differdange, Luxembourg), Khatija Bhadur (University of Applied Sciences, Differdange, Luxembourg); MALTA: Dario Silvestri (Asomi College of Malta, Malta), Valter Pagani (Asomi College of Malta, Malta), Umberto Ciriello (Asomi College of Malta, Malta), Mirko Mazzolani (Asomi College of Malta, Malta).

## References

[1] Chaput J-P Willumsen J Bull F Chou R Ekelund U Firth J. 2020 WHO guidelines on physical activity and sedentary behaviour for children and adolescents aged 5–17 years: summary of the evidence. Int J Behav Nutr Phys Act. (2020) 17(1):141. 10.1186/s12966-020-01037-z 33239009 PMC7691077

[2] Kang C Li L Huang X Jia F. Physical activity, sedentary behavior, and adolescent health: a narrative review. Front Public Health. (2026) 14:1809745. 10.3389/fpubh.2026.1809745 42110278 PMC13149281

[3] Barnett TA Kelly AS Young DR Perry CK Pratt CA Edwards NM. Sedentary behaviors in today’s youth: approaches to the prevention and management of childhood obesity: a scientific statement from the American Heart Association. Circulation. (2018) 138(11):e142–59. 10.1161/CIR.0000000000000591 30354382

[4] Miatke A Olds T Maher C Fraysse F Dumuid D. Cross-sectional and longitudinal associations between sleep, sedentary behaviour and physical activity with adiposity and cardio-respiratory fitness in school-aged children: a compositional data analysis. J Act Sedentary Sleep Behav. (2025) 4(1):11. 10.1186/s44167-025-00082-y 40646662 PMC12247391

[5] Carson V Hunter S Kuzik N Gray CE Poitras VJ Chaput J-P Saunders TJ Katzmarzyk PT Okely AD Connor Gorber S Kho ME Sampson M Lee H Tremblay MS, Systematic review of sedentary behaviour and health indicators in school-aged children and youth: an update. Appl Physiol Nutr Metab. (2016) 41(6 Suppl. 3):S240–65. 10.1139/apnm-2015-0630 27306432

[6] Wilhite K Booker B Huang B-H Antczak D Corbett L Parker P. Combinations of physical activity, sedentary behavior, and sleep duration and their associations with physical, psychological, and educational outcomes in children and adolescents: a systematic review. Am J Epidemiol. (2023) 192(4):665–79. 10.1093/aje/kwac212 36516992 PMC10089066

[7] Westerbeek H Eime R. The physical activity and sport participation framework—a policy model toward being physically active across the lifespan. Front Sports Act Living. (2021) 3:608593. 10.3389/fspor.2021.608593 34027402 PMC8138121

[8] Calcaterra V Zuccotti G. Physical exercise as a non-pharmacological intervention for attenuating obesity-related complications in children and adolescents. Int J Environ Res Public Health. (2022) 19(9):5046. 10.3390/ijerph19095046 35564441 PMC9100328

[9] Bell AC Richards J Zakrzewski-Fruer JK Smith LR Bailey DP. Sedentary behaviour—a target for the prevention and management of cardiovascular disease. Int J Environ Res Public Health. (2022) 20(1):532. 10.3390/ijerph20010532 36612852 PMC9819496

[10] Thivel D Tremblay A Genin PM Panahi S Rivière D Duclos M. Physical activity, inactivity, and sedentary behaviors: definitions and implications in occupational health. Front Public Health. (2018) 6:288. 10.3389/fpubh.2018.00288 30345266 PMC6182813

[11] Tremblay MS Aubert S Barnes JD Saunders TJ Carson V Latimer-Cheung AE Chastin SFM Altenburg TM Chinapaw MJM, on behalf of SBRN Terminology Consensus Project Participants, Tremblay MS Aubert S Barnes JD Saunders TJ Carson V, Sedentary behavior research network (SBRN)—terminology consensus project process and outcome. Int J Behav Nutr Phys Act. (2017) 14(1):75. 10.1186/s12966-017-0525-8 28599680 PMC5466781

[12] Bull FC Al-Ansari SS Biddle S Borodulin K Buman MP Cardon G. World health organization 2020 guidelines on physical activity and sedentary behaviour. Br J Sports Med. (2020) 54(24):1451–62. 10.1136/bjsports-2020-102955 33239350 PMC7719906

[13] Herold F Mavilidi MF Vazou S Benzing V Martins R Bähr F. A time-centred perspective on classroom-based physical activity. Eur J Educ. (2026) 61(3):e70752. 10.1111/ejed.70752

[14] Amor-Barbosa M Ortega-Martínez A Carrasco-Uribarren A MC B-C. Active school-based interventions to interrupt prolonged sitting improve daily physical activity: a systematic review and meta-analysis. Int J Environ Res Public Health. (2022) 19(22):15409. 10.3390/ijerph192215409 36430128 PMC9693257

[15] Hasson RE Tam T Myers L Schwartz A Li A. Improving usability of the interrupting prolonged sitting with activity virtual teacher training modules: case study. JMIR Hum Factors. (2026) 13:e83427. 10.2196/83427 42013411 PMC13099026

[16] Ferrari I Bretz K Kress J Niederberger L Schuler Braunschweig P. Sports activities in extended education—their leading role for educational development. Front Educ. (2025) 10:1536664. 10.3389/feduc.2025.1536664

[17] Schmidt M Benzing V Kamer M. Classroom-based physical activity breaks and children’s attention: cognitive engagement works!. Front Psychol. (2016) 4:7. 10.3389/fpsyg.2016.01474 PMC5047899 27757088

[18] Watson A Timperio A Brown H Best K Hesketh KD. Effect of classroom-based physical activity interventions on academic and physical activity outcomes: a systematic review and meta-analysis. Int J Behav Nutr Phys Act. (2017) 14(1):114. 10.1186/s12966-017-0569-9 28841890 PMC5574081

[19] Wagemakers A Elkhuizen S Bindels A Wachter M Niewold D Derkzen M. A participatory systems approach in community health promotion: lessons learnt from an overall evaluation of a program to reduce health inequities. Eval Program Plann. (2025) 113:102695. 10.1016/j.evalprogplan.2025.102695 40884876

[20] Karlander R Geidne S. Health promotion meets sustainable movement-based education in school—a “walking school” initiative in Sweden. Health Promot Int. (2025) 40(1):daaf006. 10.1093/heapro/daaf006 39945276 PMC11822375

[21] Zuccotti G Ascani FB Romeo AF Calcaterra V Team PODC. Beyond sport to combat childhood obesity: educating, engaging, and preventing through an integrated school-based campaign grounded in the values of olympism. Front Endocrinol. (2025) 16:1636728. 10.3389/fendo.2025.1636728 PMC12283327 40704149

[22] Annelin A Boström GO. Interdisciplinary perspectives on sustainability in higher education: a sustainability competence support model. Front Sustain. (2024) 5:1416498. 10.3389/frsus.2024.1416498

[23] Custodio-Ferrando A Cabero-Fayos I. Transdisciplinarity in education: a narrative review of its didactic validity and contributions to sustainable learning. Sustainability. (2025) 17(22):10320. 10.3390/su172210320

[24] Burnard P Colucci-Gray L Sinha P. Transdisciplinarity: letting arts and science teach together. Curric Perspect. (2021) 41(1):113–8. 10.1007/s41297-020-00128-y

[25] Talevski J Wong Shee A Rasmussen B Kemp G Beauchamp A. Teach-back: a systematic review of implementation and impacts. Mathes T, editor. PLoS One. (2020) 15(4):e0231350. 10.1371/journal.pone.0231350 32287296 PMC7156054

[26] Câmpean A Bocoș M Roman A Rad D Crișan C Maier M. Examining teachers’ perception on the impact of positive feedback on school students. Educ Sci. (2024) 14(3):257. 10.3390/educsci14030257

[27] Gan Z An Z Liu F. Teacher feedback practices, student feedback motivation, and feedback behavior: how are they associated with learning outcomes? Front Psychol. (2021) 12:697045. 10.3389/fpsyg.2021.697045 34234729 PMC8257033

[28] Wang C Zhang P Zhu Y Li J Yang Y Tan X. A theoretical model of sports and health integration to promote active health. BMC Public Health. (2025) 25(1):1039. 10.1186/s12889-025-22237-x 40102802 PMC11917084

[29] Liao CC Hsu CH Kuo KP Luo YJ Kao CC. Ability of the sport education model to promote healthy lifestyles in university students: a randomized controlled trial. Int J Environ Res Public Health. (2023) 20(3):2174. 10.3390/ijerph20032174 36767543 PMC9915953

[30] Bachouri-Muniesa H Lhuisset L Aibar A Fabre N Asún-Dieste S Bois JE. Dissemination, implementation, and evaluation of an effective school-based intervention to promote physical activity in adolescents: a study protocol. Behav Sci. (2023) 13(4):290. 10.3390/bs13040290 37102804 PMC10135457

