# Supplementary material for: From sport to everyday movement: a school-based “DON'T STAY STILL!” initiative to address sedentary behavior in children
Source: Front Sports Act Living. 2026 Oct 14;8:1979089. doi: 10.3389/fspor.2026.1979089 (PMC13714929; doi:10.3389/fspor.2026.1979089)
Supplement: Supplementary file 1 [file Datasheet1.pdf]

PoDiaCar

Co-funded by the European Union

Per promuovere uno stile di vita attivo e prevenire sedentarietà, obesità e malattie correlate.

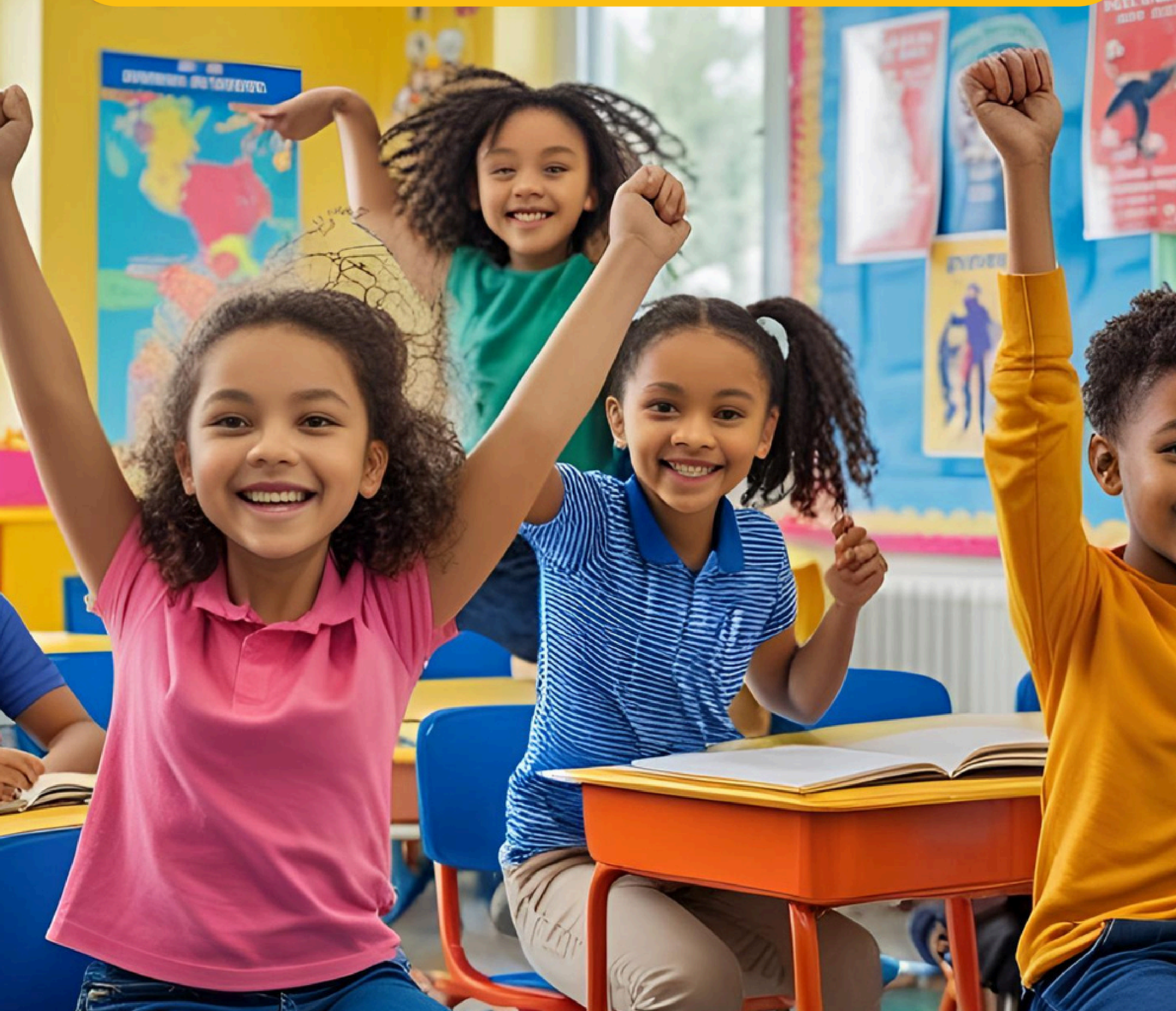

NON RESTARE FERMO!

Indice

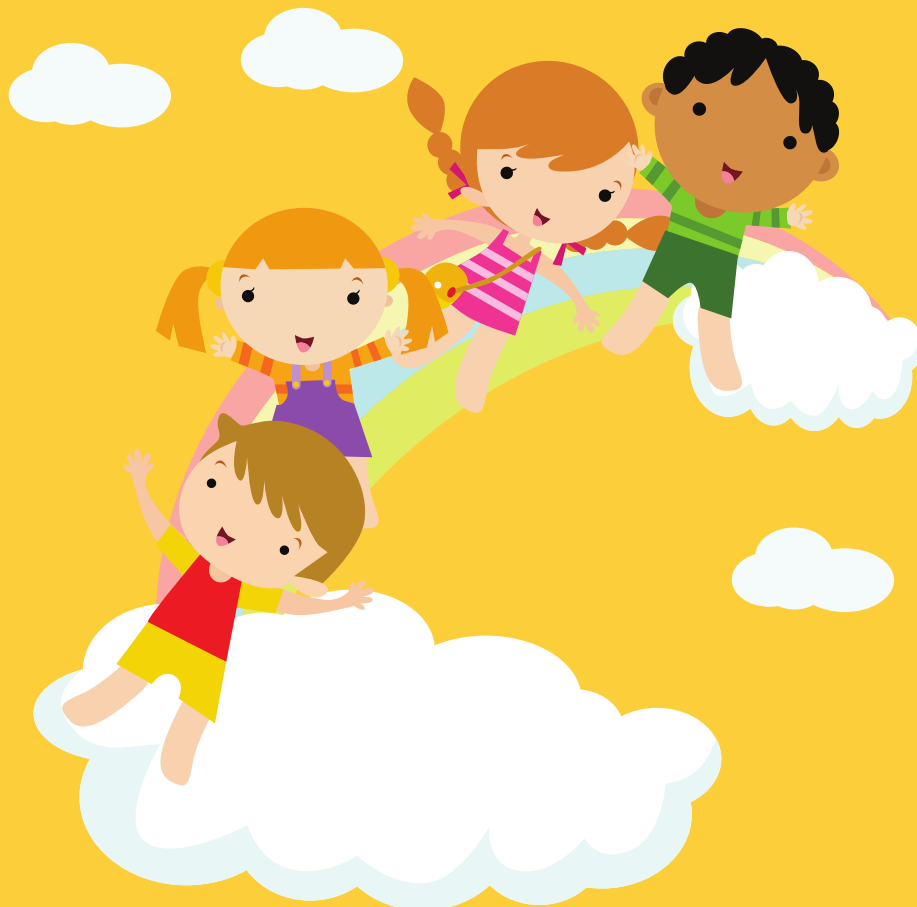

03 Introduzione

04 La sedentarietà

05 I rischi della sedentarietà sulla salute

06 I benefici dell'esercizio fisico

07 La promozione di uno stile di vita attivo nel contesto scolastico

08 "Momenti di movimento": spunti per pause attive in aula

10 Educare al benessere, all'inclusione e alla partecipazione

11 La piramide dell'attività motoria

12 Strategie per uno stile di vita attivo in famiglia

13 Attività fisica e sana alimentazione

14 Conclusioni

Co-funded by the European Union

UNIVERSITÀ DEGLI STUDI DI MILANO

UNIVERSITÀ DI PAVIA

Ospedale dei Bambini Vittore Buzzi

Sistema Socio Sanitario Regione Lombardia ASST Fatebenefratelli Sacco

Introduzione

Il progetto "**PODiaCar: Combattere l'Obesità Pediatrica. Da uno strumento predittivo per il rischio di diabete di tipo 2 e malattie cardiovascolari a programmi educativi per la salute"**, è un progetto co-finanziato dalla Comunità Europea (EU4H-2022-Pj-3-EU4H-PJG), che vuole proporre un modello multidimensionale per combattere l'obesità.

L'**obesità pediatrica** è una condizione caratterizzata da un eccessivo accumulo di grasso corporeo tale da compromettere la salute a breve e lungo termine.
L'obesità pediatrica, rappresenta un **problema di salute pubblica** in crescita a livello globale e particolarmente preoccupante nei paesi industrializzati. La prevalenza della malattia è aumentata significativamente negli ultimi decenni, a causa di diversi fattori ambientali, comportamentali e genetici.

In **Italia**, secondo quanto riportato da OKkio alla SALUTE, indagine condotta dall'Istituto Superiore di Sanità (ISS) in collaborazione con il Ministero dell'Istruzione e del Merito e con tutte le Regioni e Provincie autonome italiane, i bambini e le bambine di 8-9 anni in sovrappeso sono il 19% e con obesità il 9,8%, inclusi bambine e bambini con obesità grave che rappresentano il 2,6%

- Modelli alimentari sbilanciati e bassi livelli di attività fisica sono i principali fattori di rischio di sviluppare obesità. Due bambini e bambine italiani su 5 non fanno una colazione adeguata al mattino, più della metà consuma una merenda abbondante a metà mattina, 1 su 4 beve quotidianamente bevande zuccherate/gassate e consuma frutta e verdura meno di una volta al giorno, la metà di loro mangia snack dolci più di 3 giorni a settimana.
- Le conseguenze dell'obesità nei bambini possono essere sia fisiche che psicologiche. In particolare, i bambini con obesità sono più a rischio di sviluppare problemi di salute come diabete di tipo 2, ipertensione, dislipidemia, apnee notturne, problemi articolari e malattie cardiovascolari.
- Inoltre, l'obesità pediatrica è associata a una maggiore probabilità di obesità in età adulta, con il rischio conseguente di sviluppare patologie croniche.
- I bambini con obesità possono inoltre essere soggetti a bullismo, discriminazione e stigmatizzazione, con effetti negativi sull'autostima, portando a depressione, ansia e problemi legati all'immagine corporea.
- Servono pertanto proposte innovative per contrastare l'obesità pediatrica e salvaguardare il benessere e la salute dei bambini.

Per sostenere stili di vita sani PODiaCar promuove una campagna di sensibilizzazione multicanale e programmi educativi per i bambini nelle scuole e in famiglia.
PODiaCar prevede altresì la creazione di algoritmi e modelli predittivi, come strumento di diagnosi precoce e prevenzione dell'obesità e delle complicanze ad essa correlata, quali diabete mellito tipo 2 e malattie cardiovascolari, nella popolazione pediatrica.

Innovazione ed educazione possono essere alleati preziosi per combattere l'obesità pediatrica.

PODiaCar
Co-funded by the European Union

LA SEDENTARIETÀ

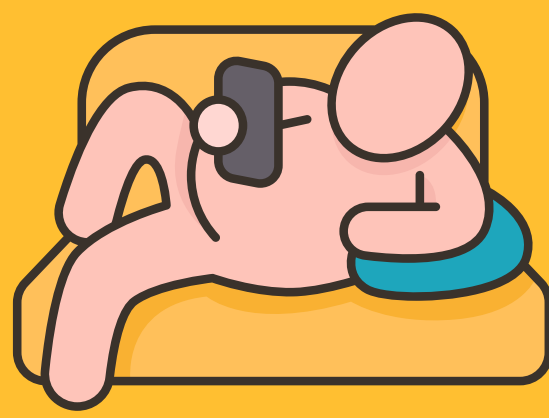

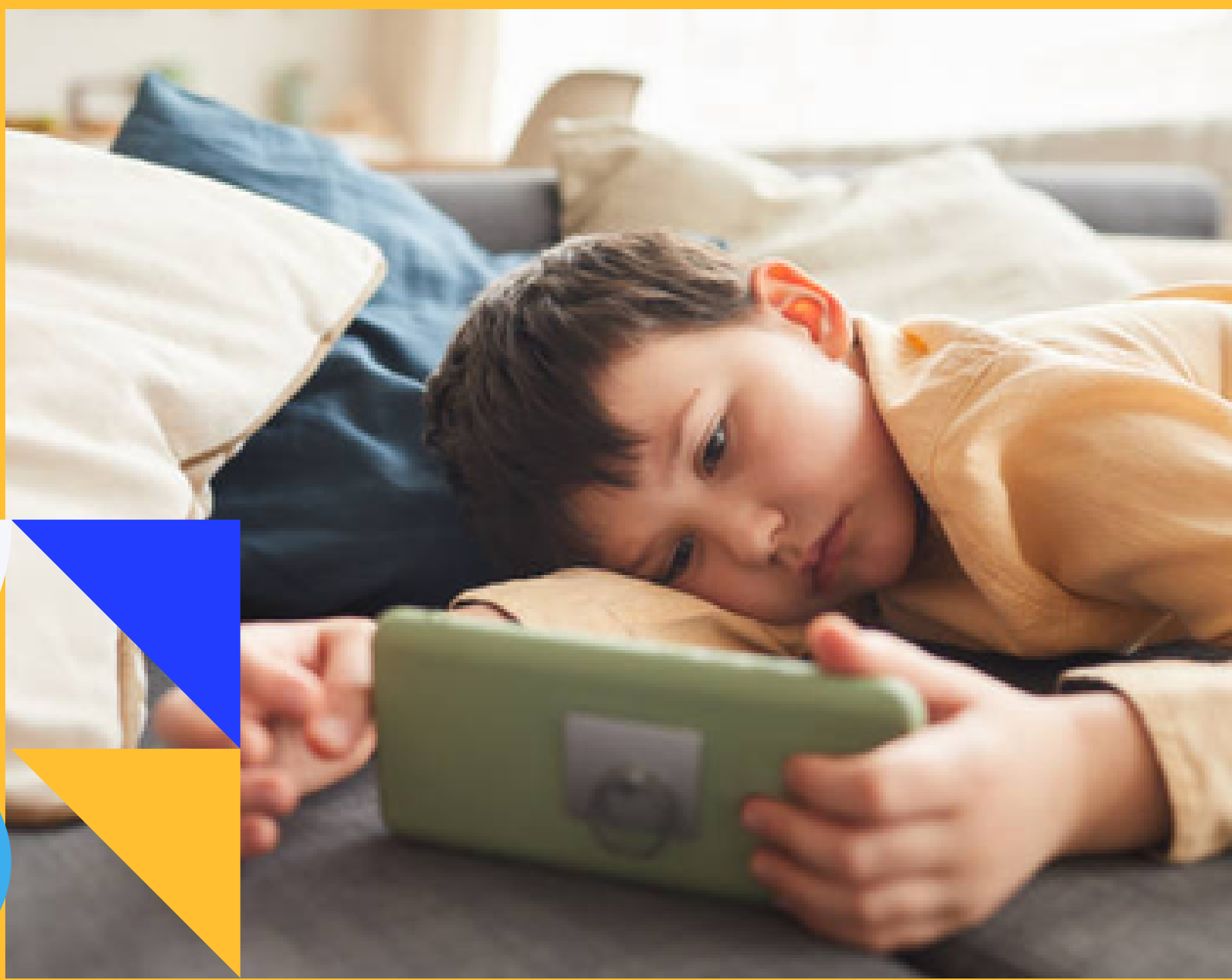

La sedentarietà si definisce come l'insieme di comportamenti caratterizzati da un basso/assente livello di attività fisica e da un ridotto dispendio energetico. Il termine ha origine dal latino "sedentarius", che indicava "colui che resta seduto", dunque una condizione statica e prolungata nel tempo, come per esempio essere seduti o distesi

La crescente diffusione di comportamenti sedentari, anche in età evolutiva, rappresenta una sfida prioritaria per il settore educativo. Purtroppo è sempre più frequente la riduzione delle ore di movimento durante l'orario scolastico ed extrascolastico. Di conseguenza questo impatta in modo negativo sulla salute creando abitudini non sane, vizi posturali, cali di energia e scarso coinvolgimento sociale di tutta la popolazione pediatrica.

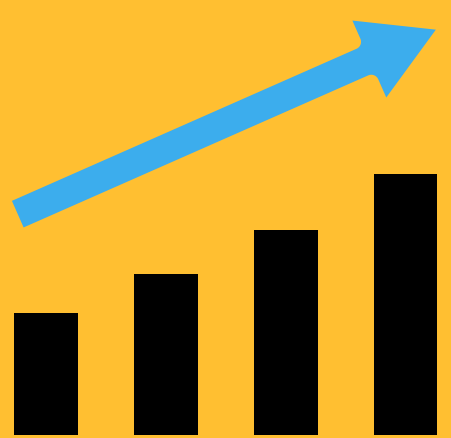

Sorveglianza PASSI 2022-2023

I dati riguardanti i bambini e ragazzi (fascia d'età dai 3 ai 18 anni) indicano che il livello di sedentarietà aumenta in maniera inversalmente proporzionale all'età. Dopo i 15 anni gli adolescenti cessano progressivamente l'attività fisica (le ragazze più dei ragazzi).

Lo sport viene sostituito con attività e giochi statici (PC, console, smartphone ecc). Dopo la fine del percorso scolastico più del 50% degli adolescenti diventa totalmente sedentario.

UNO STILE DI VITA SEDENTARIO PUÒ CAUSARE GRAVI PROBLEMI DI SALUTE A LUNGO TERMINE

I RISCHI DELLA SEDENTARIETÀ

La **sedentarietà** impatta in modo negativo su molteplici aspetti durante l'età evolutiva

La sedentarietà rappresenta un potenziale fattore di rischio per la salute fisica e psicologica del giovane con conseguenze anche nell'età adulta

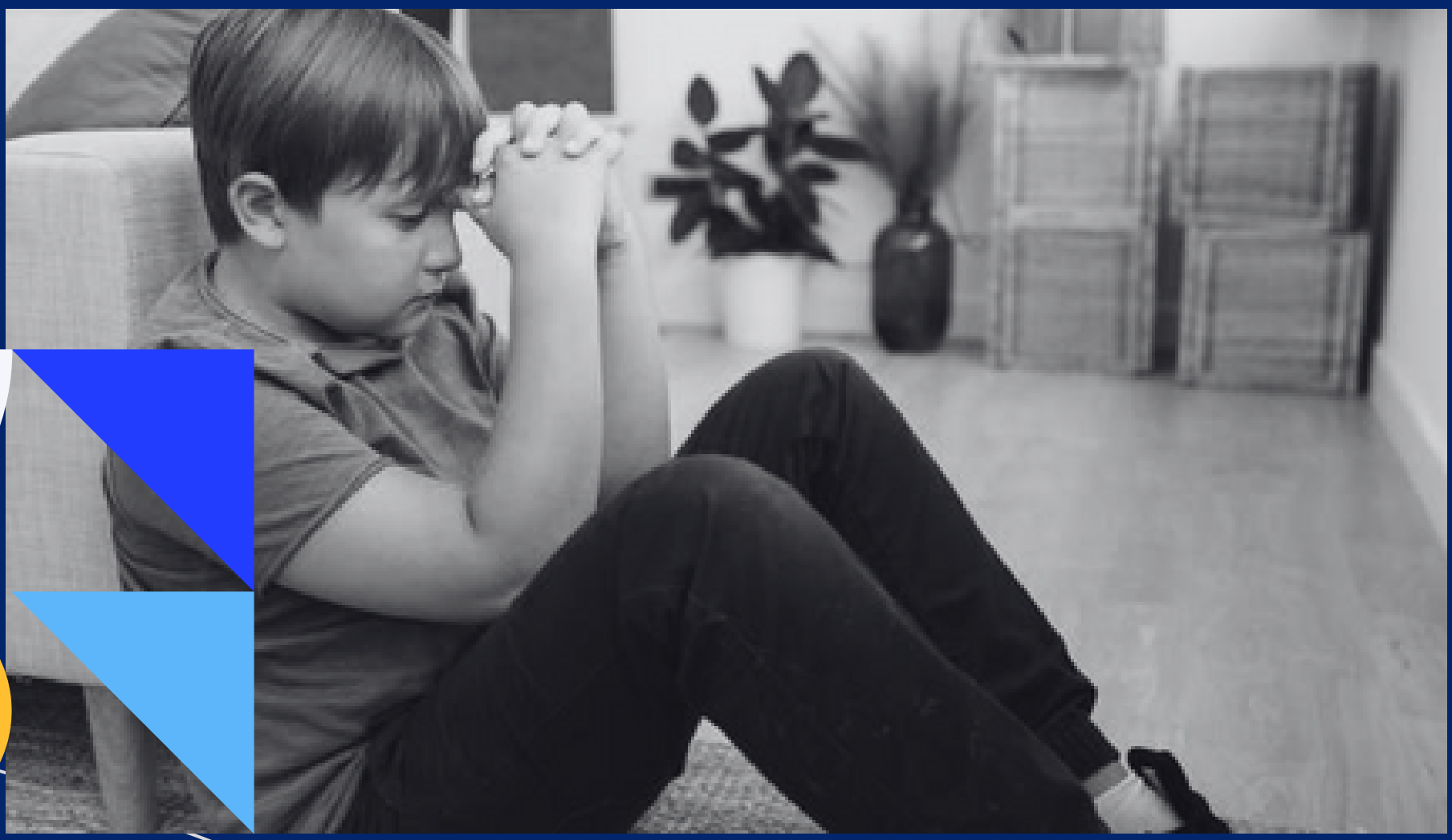

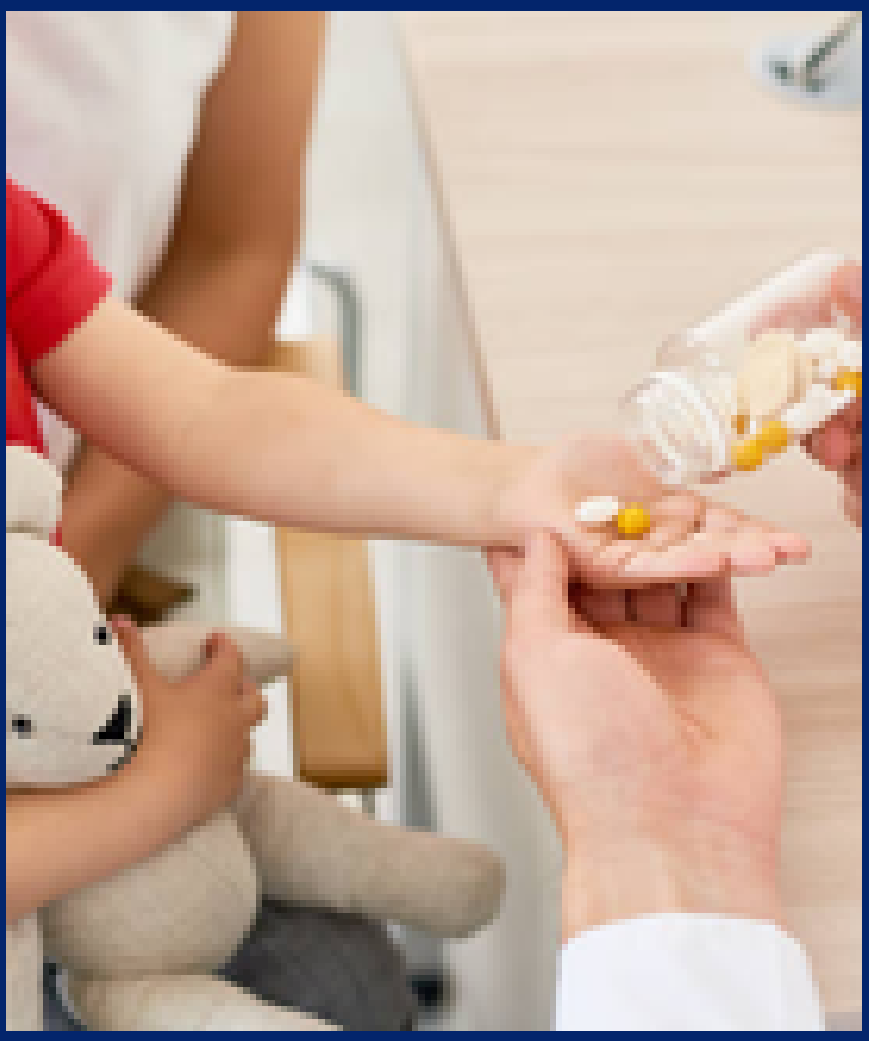

**Rischi FISIOLOGICI**	Incremento della **massa grassa** e insorgenza di **sovrappeso/obesità**
	**Riduzione della capacità cardiorespiratoria** e della resistenza muscolare
	Compromissione dell'**apparato muscolo-scheletrico** e della postura
	Maggiore incidenza di **malattie croniche** in età adulta
**Rischi PSICOLOGICI**	Diminuzione delle **funzioni esecutive**, della capacità di **attenzione** e della **memoria di lavoro**
	Maggiore predisposizione a **disturbi d'ansia, stress e sintomi depressivi**
	Riduzione del livello di **motivazione scolastica** e dell'**autoefficacia percepita**
	Compromissione dell'**equilibrio emotivo**, con aumento di comportamenti oppositivi o regressivi
**Rischi PSICOSOCIALI**	**Isolamento relazionale** e diminuzione della capacità di interazione tra pari
	**Esclusione da contesti sportivi o ludico-motori**, in particolare in presenza di disabilità o fragilità
	Riduzione del **senso di appartenenza** al gruppo e della **partecipazione attiva alla vita scolastica**
	Mancanza di stimolo delle **competenze sociali e cooperative**

I BENEFICI DELL'ESERCIZIO FISICO

UN PILASTRO FONDAMENTALE DELLA PREVENZIONE

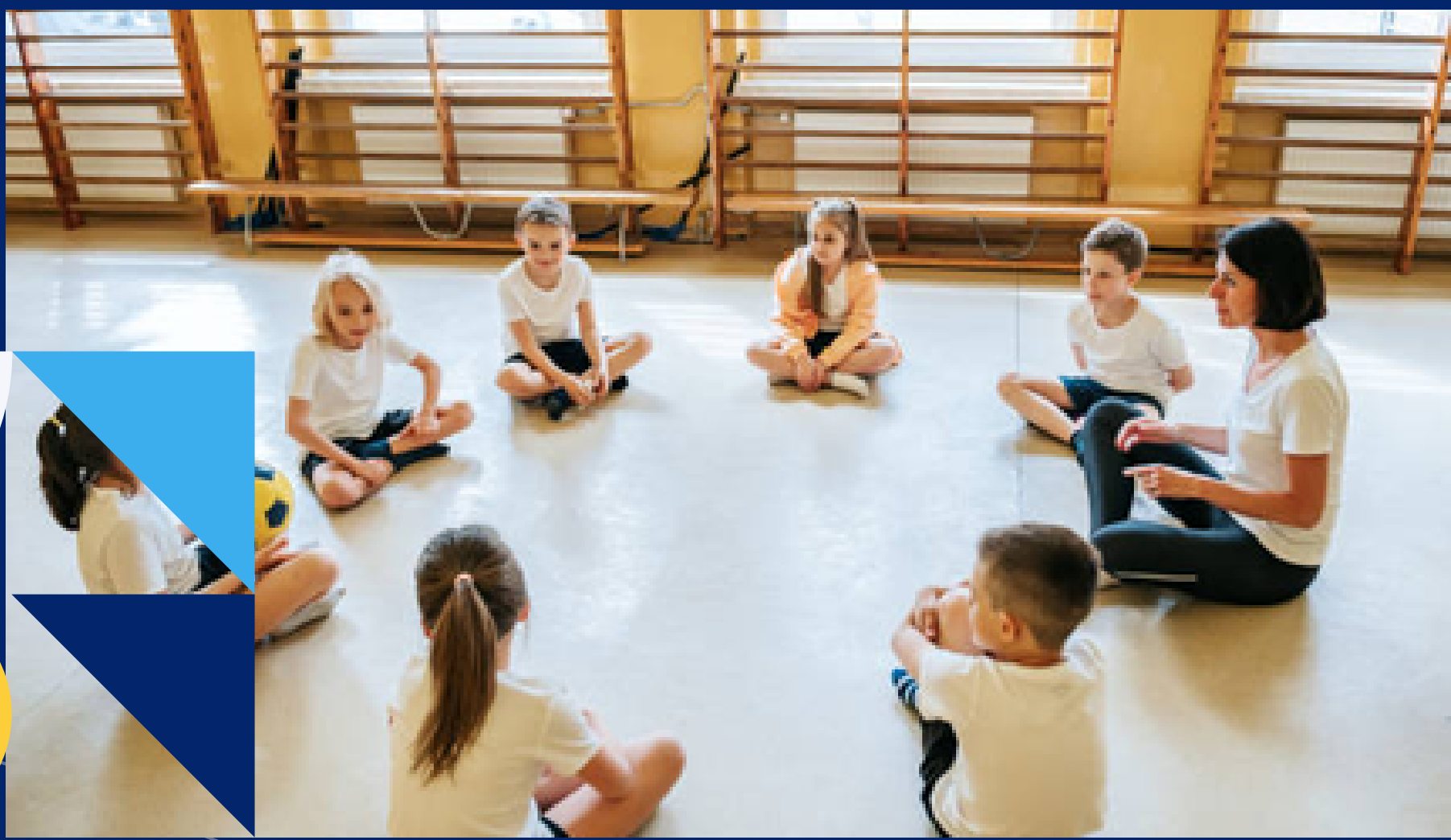

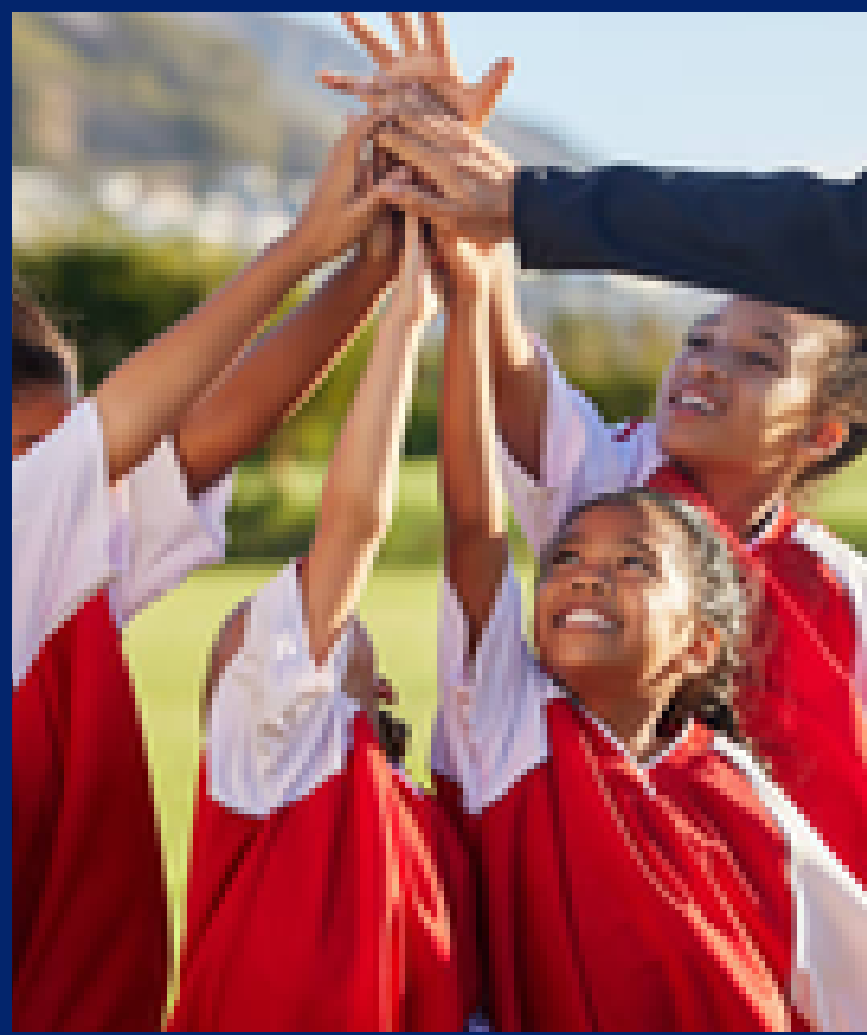

Numerose evidenze scientifiche confermano che la pratica regolare di esercizio fisico riduce in modo significativo il rischio di sviluppare patologie cardiovascolari , metaboliche, psicologiche, nonché malattie oncologiche e reumatiche

Lo sviluppo delle capacità condizionali — resistenza, forza, velocità e flessibilità — riveste un ruolo centrale nella promozione della salute fisica

- La **resistenza aerobica** migliora il profilo cardiovascolare, metabolico e respiratorio. Inoltra aumenta la capacità di mantenere uno sforzo nel tempo
- La **forza** contribuisce all'aumento di massa magra, alla stabilità posturale e riduce il rischio di infortuni
- La **velocità** è funzionale all'efficienza motoria nei gesti rapidi, è un mezzo per potenziare la reattività e la coordinazione.
- La **flessibilità** garantisce una buona mobilità articolare, utile nei gesti

Lo sviluppo di **strategie** che mirano ad **aumentare la diffusione dell'attività fisica**, attraverso l'attivazione di interventi efficaci, è un obiettivo di sanità pubblica che può essere raggiunto solo attraverso politiche sanitarie mirate, condivisione di obiettivi e individuazione delle responsabilità.

LA PROMOZIONE DI UNO STILE DI VITA ATTIVO NEL CONTESTO SCOLASTICO

La **scuola** riveste un ruolo fondamentale nella promozione di **stili di vita sani e attivi**. Da diversi anni in Italia sono stati adottati diversi programmi e iniziative di sperimentazione per promuovere l'**alfabetizzazione motoria**, il **movimento** e lo **sport** in classe. Infatti l'attività fisica in tutte le sue forme contribuisce anche a migliorare la qualità dell'apprendimento

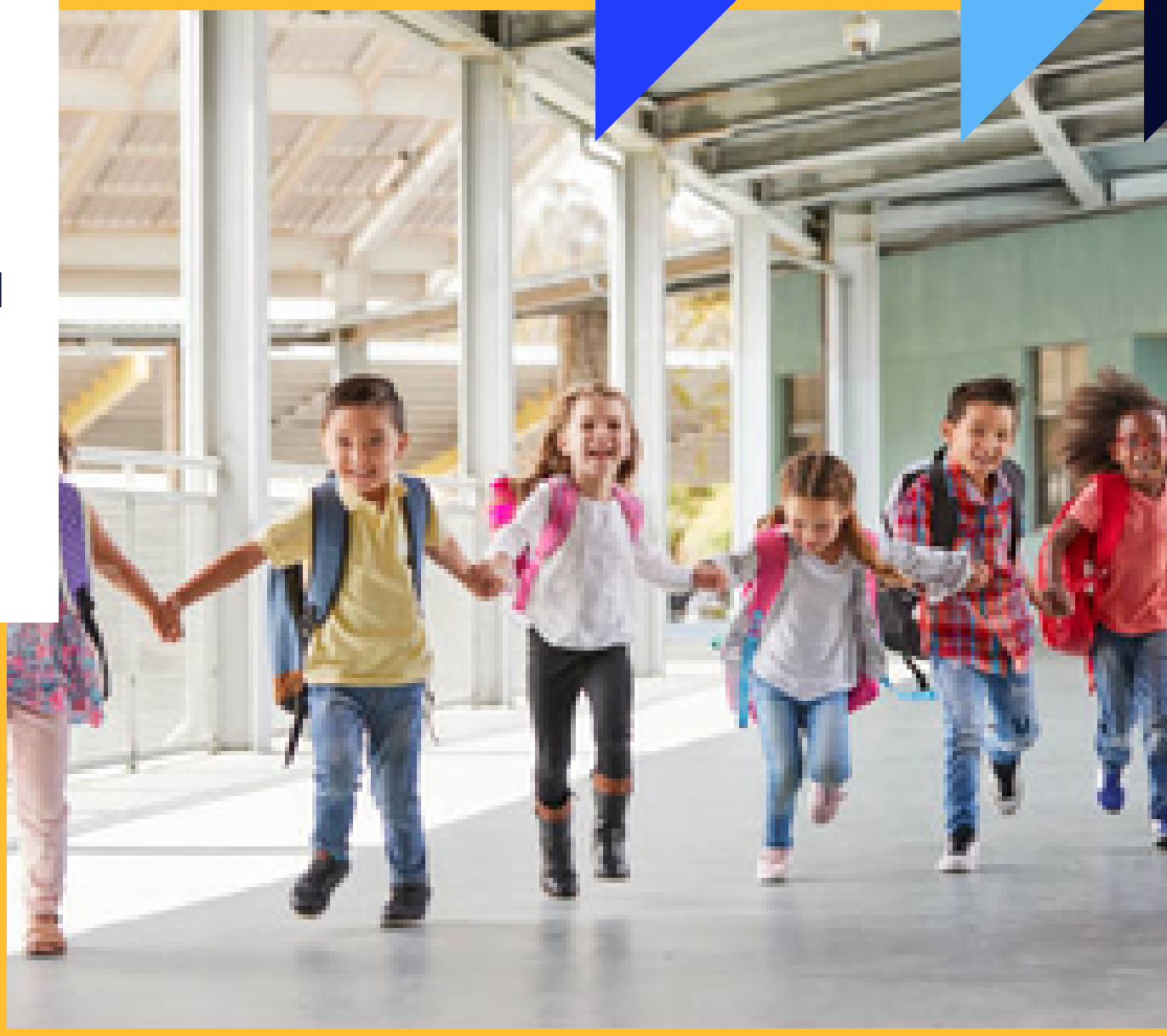

EDUCAZIONE FISICA, UNO STRUMENTO PER...

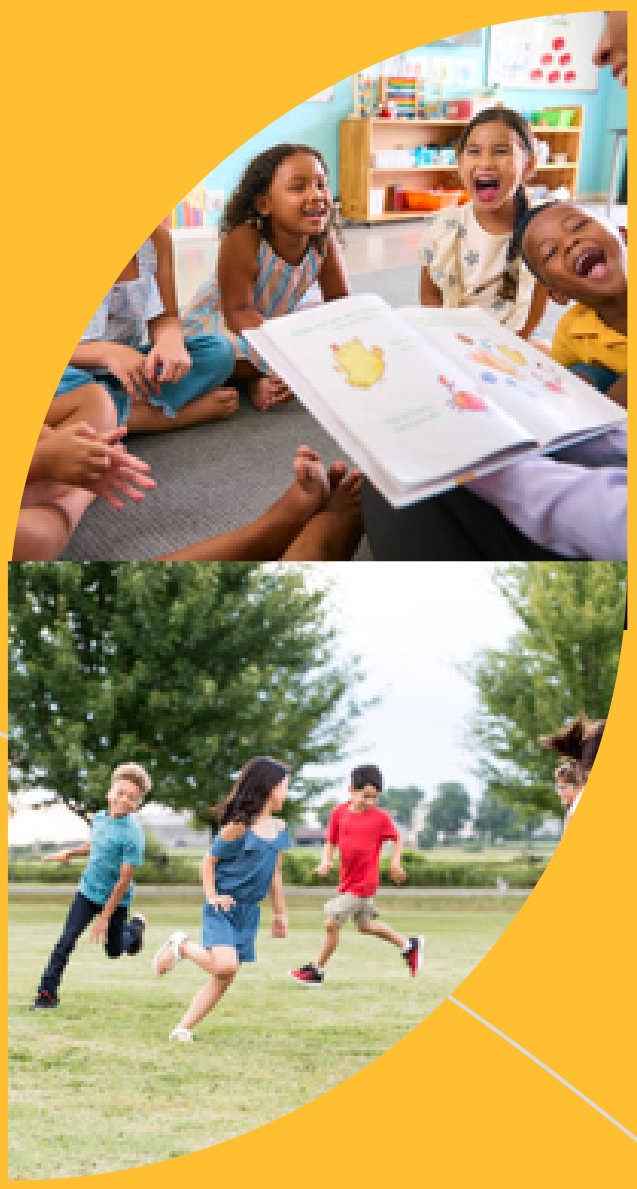

- lo sviluppo della consapevolezza di sé a livello corporeo e motorio,
- promuovere lo sviluppo di stili di vita sani come lo svolgimento di attività fisica regolare e l'adozione di abitudini alimentari salutari,
- lo sviluppo di conoscenze, abilità e competenze motorie,
- uno sviluppo integrato fisico e cognitivo, per l'inclusione e la partecipazione di ogni persona alle attività attraverso i necessari adattamenti,
- stimolare la partecipazione attiva degli studenti e la collaborazione nel pieno rispetto di sé, degli altri e delle regole (fair play)

ADOTTARE UN APPROCCIO GLOBALE CHE COINVOLGE L'INTERA COMUNITÀ EDUCANTE (INSEGNANTI, FAMIGLIE, PERSONALE SCOLASTICO) È ESSENZIALE PER RENDERE SOSTENIBILE E CONTINUATIVA LA PROMOZIONE DEL MOVIMENTO

“Momenti di movimento”

SPUNTI PER PAUSE ATTIVE IN AULA

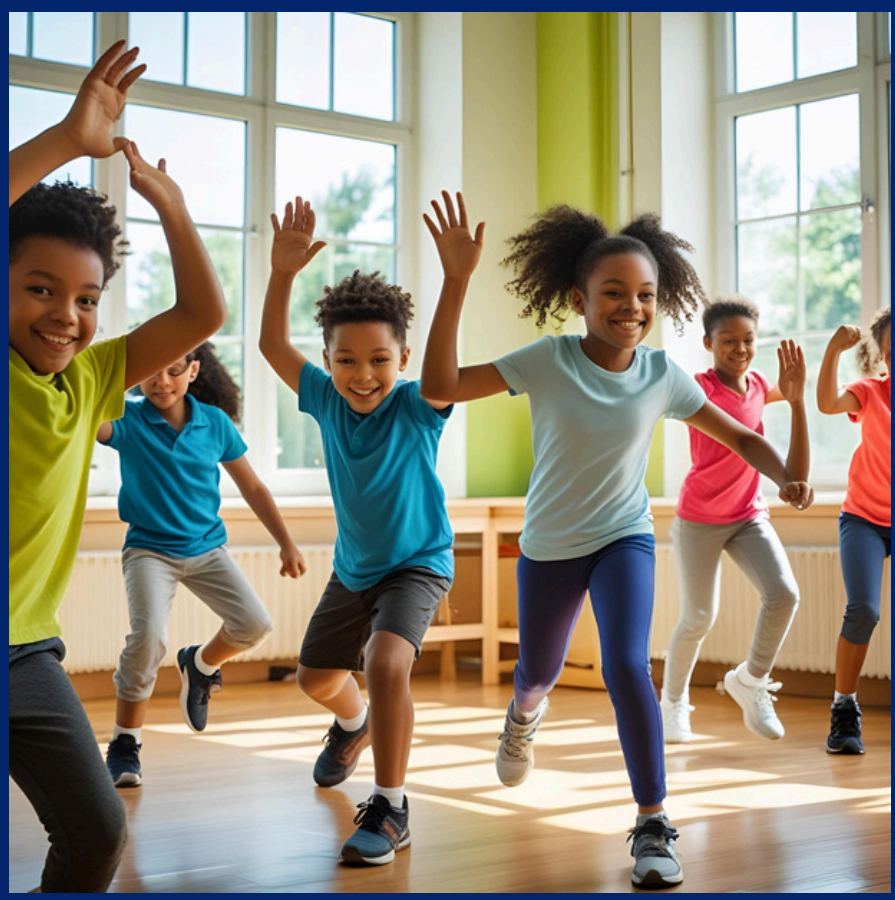

Le **pause attive** rappresentano una strategia semplice ed efficace per **interrompere la sedentarietà** e **migliorare la concentrazione** durante l’orario scolastico curriculare

Nello specifico, promuovere brevi momenti di attività motoria guidata durante la lezione oppure nel cambio dell’ora rappresenta una sana abitudine per contrastare la sedentarietà

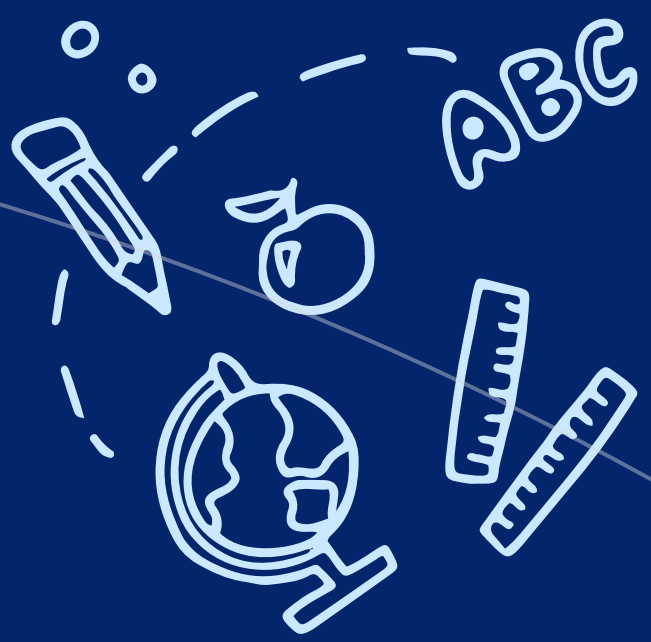

Questi intervalli attivi contribuiscono non solo a migliorare l’efficienza dell’apprendimento, ma anche a rafforzare le dinamiche relazionali tra pari e tra alunni e docenti. Favoriscono inoltre l’empatia, l’inclusione, la creatività, la capacità di risoluzione dei problemi e il senso di appartenenza alla comunità scolastica

MOMENTI DI MOVIMENTO - proposte da eseguire in piedi/ seduti

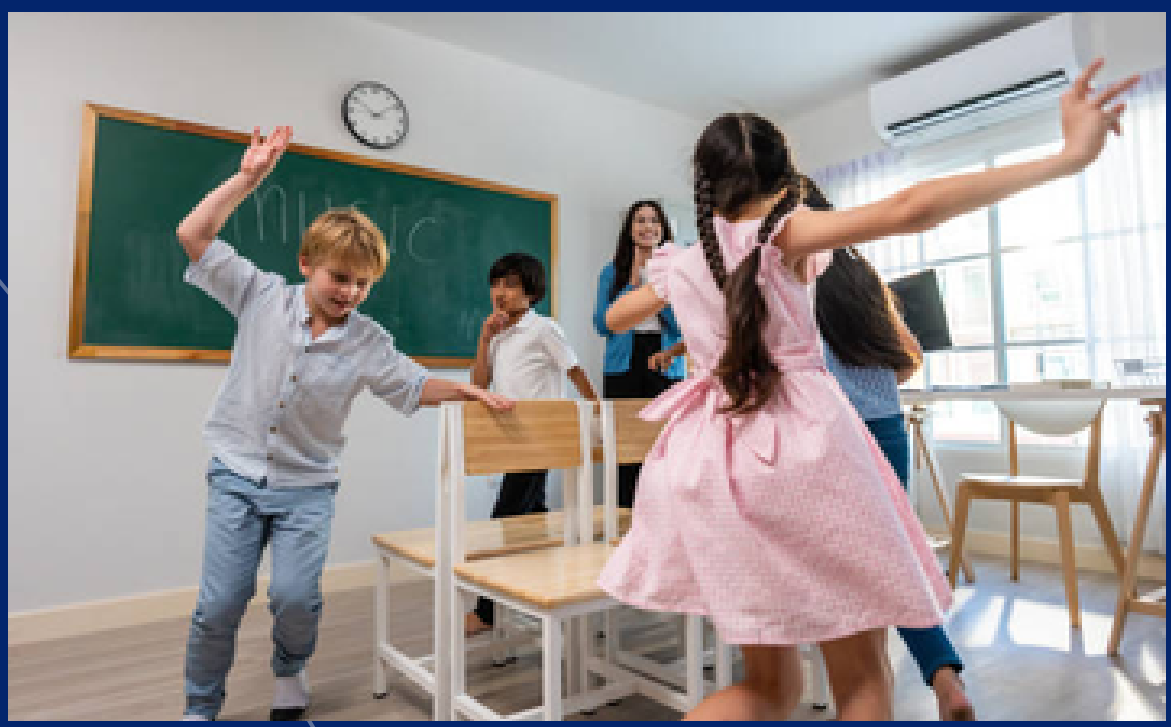

- **Esercizi di stretching**: Allungamenti semplici e guidati da eseguire in piedi accanto al banco o in cerchio, per sciogliere le tensioni muscolari e migliorare la postura
- **Giochi motori:** attività ludiche che coinvolgono il corpo in modo creativo, come mimare animali, fare piccoli salti, camminare in equilibrio su una linea immaginaria
- **Brevi sequenze motorie a ritmo**: movimenti eseguiti da tutta la classe seguendo una musica o una conta: semplici coreografie, battiti di mani e piedi, piccoli esercizi dinamici
- **Camminate all’interno o all’esterno dell’aula:** passeggiate guidate nel corridoio, nel cortile o anche semplicemente intorno all’aula

“Momenti di movimento”

UNO STRUMENTO PER POTENZIARE L’APPRENDIMENTO SCOLASTICO

“Momenti di movimento” sono particolarmente indicati quando sono ideati in un’**ottica multidisciplinare** poiché favoriscono l’apprendimento trasversale, stimolano il coinvolgimento globale dell’alunno e promuovono una didattica dinamica

Gli esempi che saranno proposti hanno l’obiettivo di favorire un apprendimento più efficace e coinvolgente grazie all’intersezione di una o più materie con l’educazione fisica.

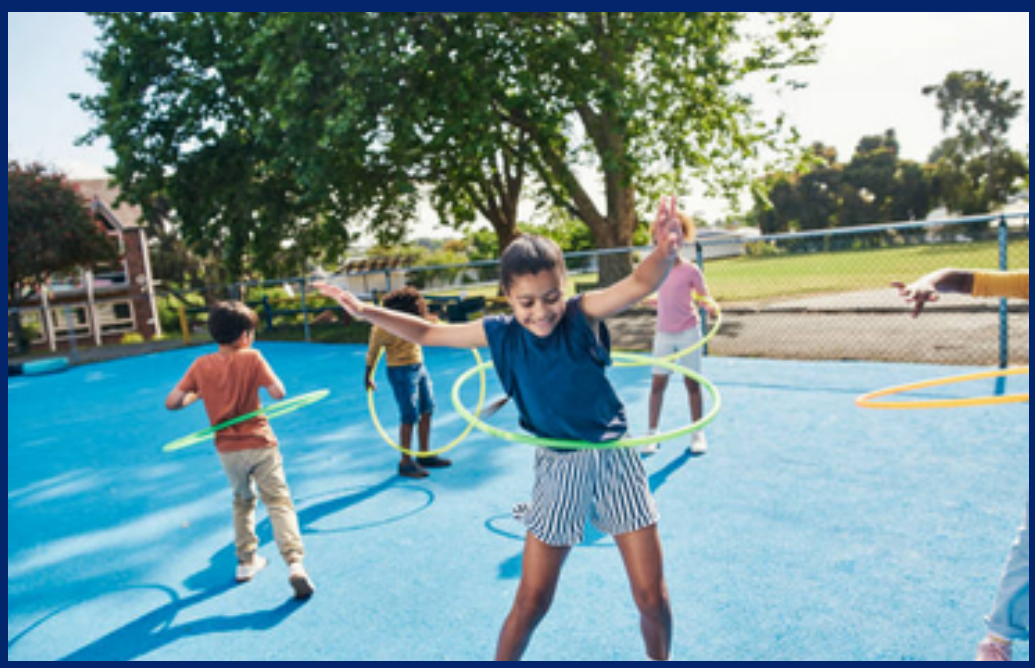

DURATA

- 5–10 minuti, da adattare all’età e al contesto
- Brevi, frequenti e regolari per maggiore efficacia

IDEE PER PAUSE ATTIVE MULTIDISCIPLINARI

- selezionare una materia
- inviduare la competenza da esercitare

ITALIANO<br>LINGUE STRANIERE	• “Salta la sillaba”: saltelli associati a sillabe o rime<br>• Mimare i verbi (es. correre, ballare, guidare...)
MATEMATICA	• Conteggio con numeri “un salto per ogni numero dispari<br>• Geometria in movimento: rappresentare figure con il corpo
SCIENZE	• Focus sul movimento della respirazione<br>• Equilibrio e leve (sollevamenti...)
ARTE	• Disegno con penna astratta enfatizzando il movimento<br>• Imitazione protagonisti di quadri e ipotesi loro movimenti

EDUCARE AL BENESSERE, ALL'INCLUSIONE E ALLA PARTECIPAZIONE

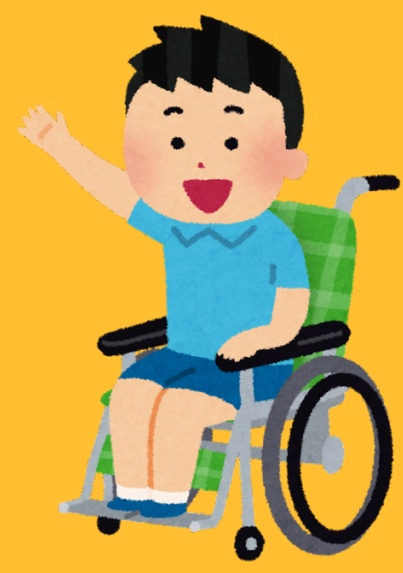

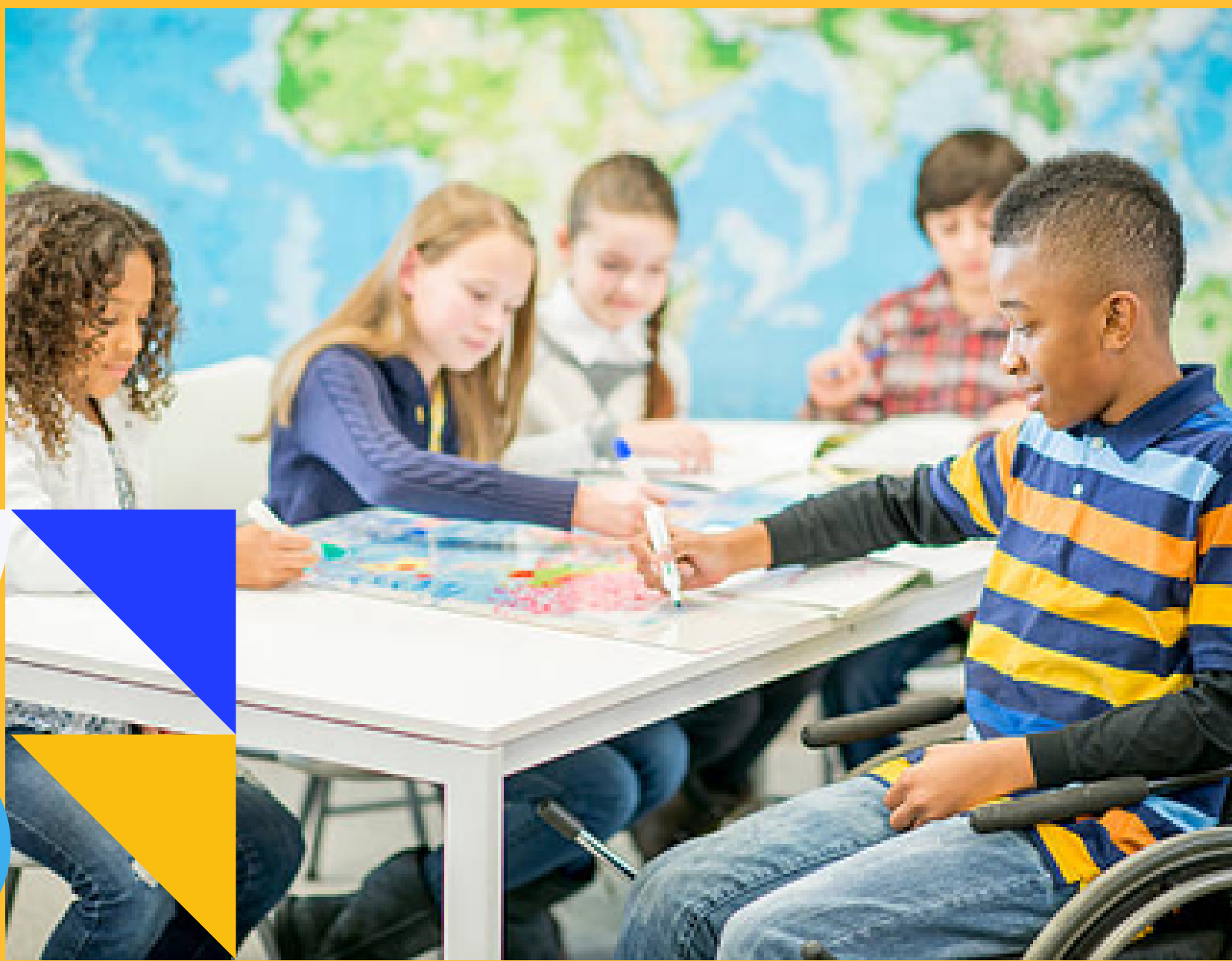

La scuola rappresenta un contesto fondamentale per promuovere uno **stile di vita attivo, inclusivo e orientato al benessere globale** degli studenti. Uno strumento didattico per favorire l'inclusione, la partecipazione e la costruzione di relazioni significative è l'attività fisica.

Per questi motivi, integrare il movimento nella quotidianità scolastica può contribuire a **valorizzare ogni alunno**, anche in presenza di disabilità, rafforzando il senso di appartenenza e contrastando le barriere che ostacolano la piena partecipazione di tutti

Educare al benessere significa promuovere nei bambini e negli adolescenti uno sviluppo armonico che integri le dimensioni fisica, emotiva, sociale e relazionale. In questo processo, l'inclusione e la partecipazione attiva rappresentano elementi essenziali per **contrastare la sedentarietà**, fenomeno sempre più diffuso anche in età evolutiva.

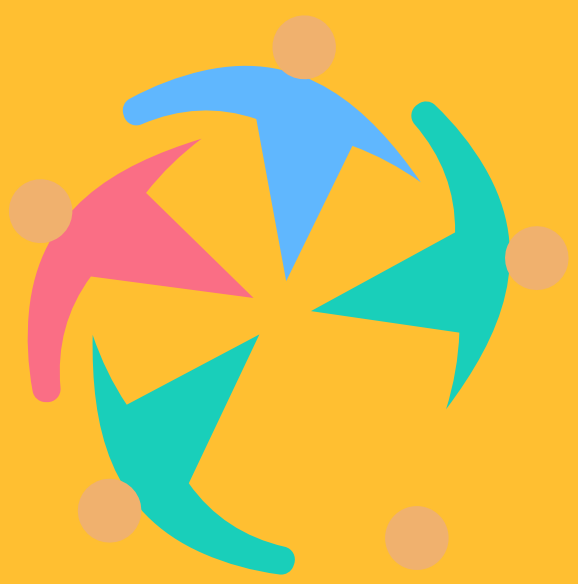

Offrire **opportunità di movimento accessibili e inclusive**, all'interno di contesti scolastici e ricreativi, consente di ridurre le disuguaglianze, valorizzare le diversità e costruire ambienti educativi che coinvolgono tutti, compresi alunni con disabilità, con difficoltà socio-economiche e linguistiche

"Un ambiente scolastico che promuove il movimento genera inclusione: contrastare la sedentarietà significa creare spazi dove tutti possono partecipare, apprendere e crescere insieme"

LA PIRAMIDE DELL’ATTIVITÀ FISICA

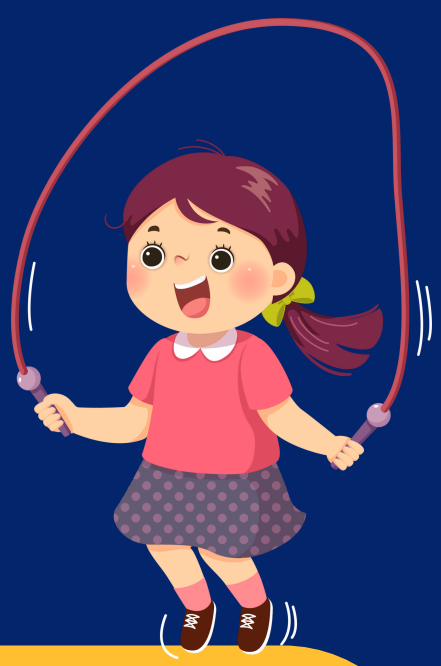

In ogni **fase dello sviluppo**, è fondamentale ridurre i periodi di inattività prolungata, con particolare attenzione al tempo trascorso davanti agli schermi (TV, tablet, smartphone). Nella popolazione di bambini

- di età inferiore ai 2 anni, si raccomanda di evitare completamente l’esposizione agli schermi;
- di età compresa tra i 2 e i 5 anni, tale utilizzo dovrebbe essere limitato a un massimo di un’ora al giorno

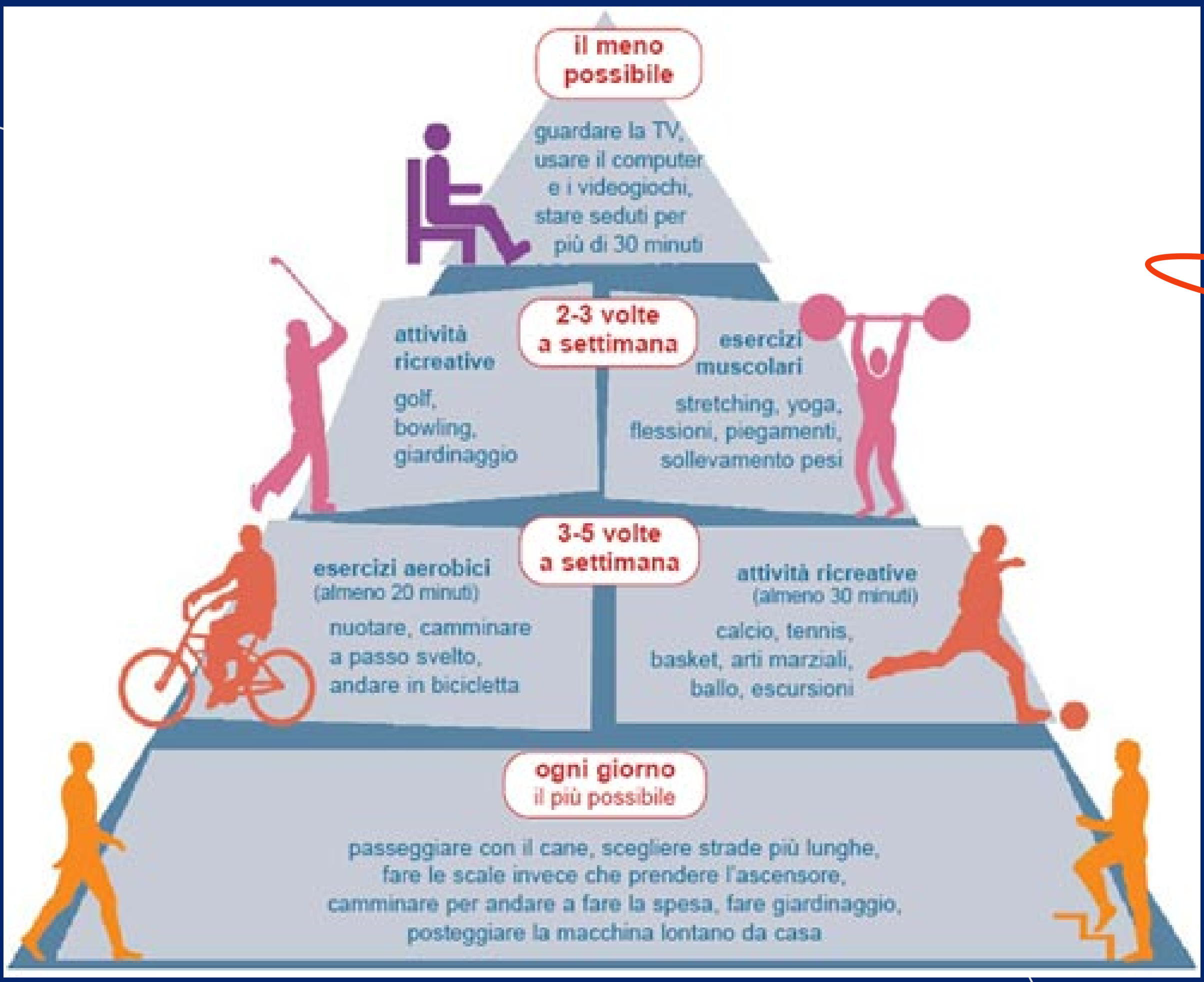

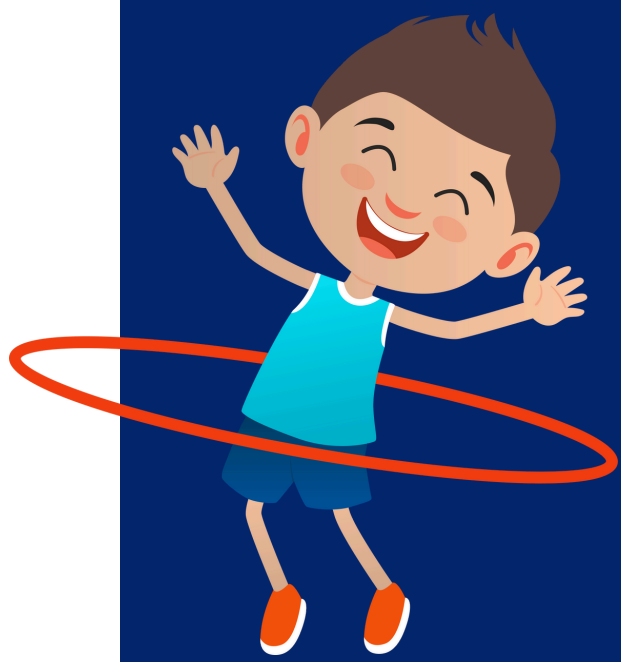

Dall’infanzia all’adolescenza, il mantenimento di **adeguati livelli di attività fisica,** sia in termini di frequenza che di intensità, combinati con un’**alimentazione equilibrata** e corretta, rappresenta un fattore determinante per una **crescita armoniosa** e per la **promozione della salute a lungo termine**

STRATEGIE PER UNO STILE DI VITA ATTIVO IN FAMIGLIA

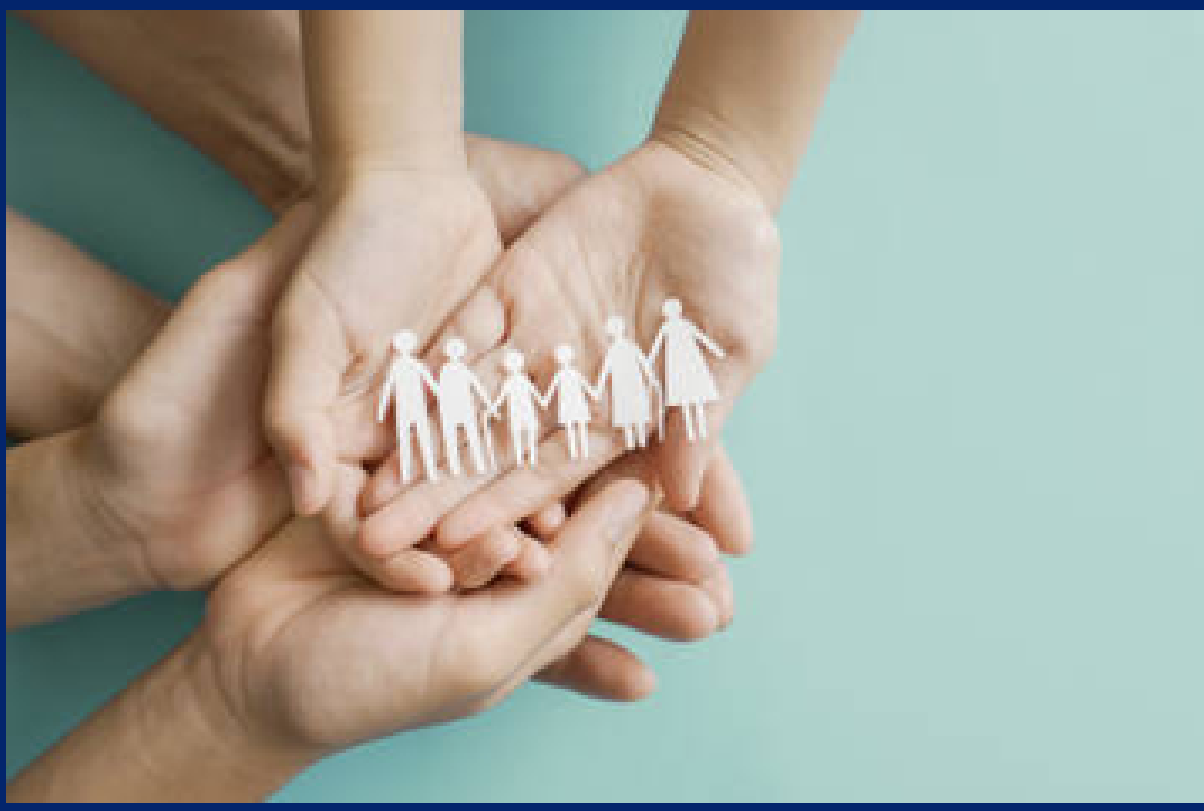

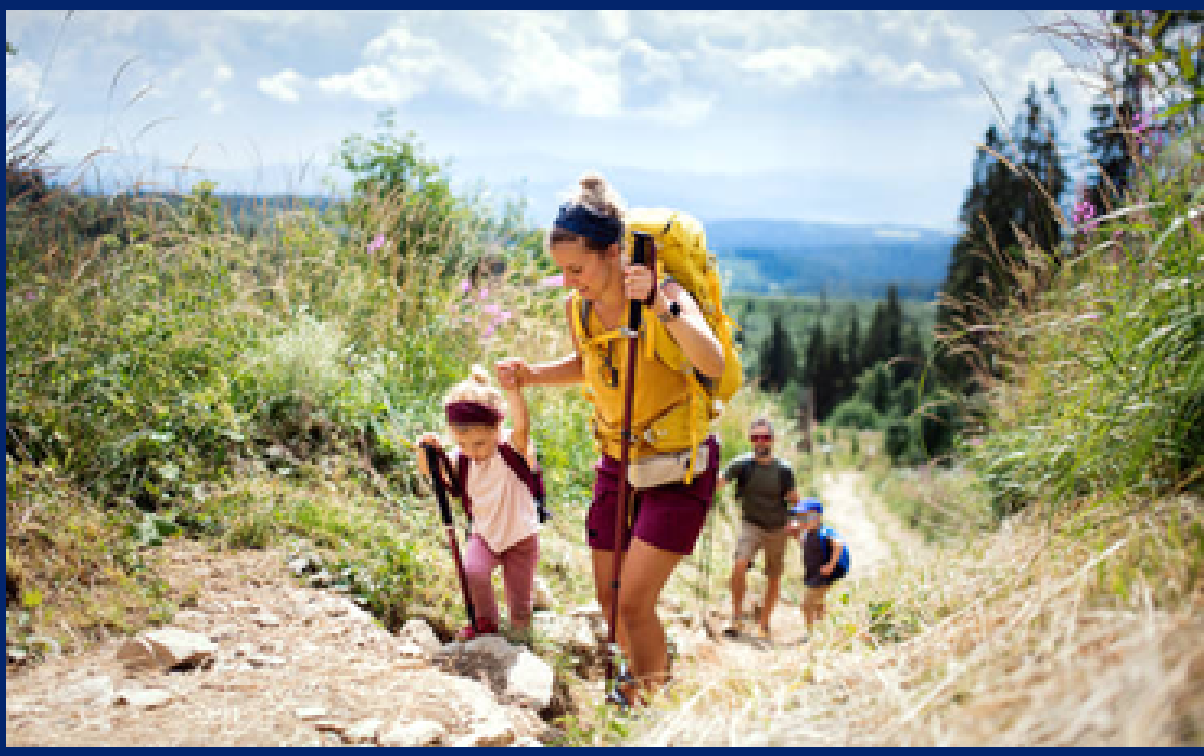

L'ESEMPIO DEI GENITORI

Per promuovere la pratica dell'attività fisica nei bambini, il ruolo attivo dei genitori risulta determinante. L'età evolutiva, infatti, è fortemente influenzata da meccanismi di apprendimento imitativo: i bambini tendono a osservare e interiorizzare i comportamenti degli adulti di riferimento, riproducendoli nelle proprie abitudini quotidiane

5 CONSIGLI ★★★★★

- **Promuovere il gioco attivo**: favorire attività ludiche all'aperto per stimolare il movimento e rafforzare la relazione genitori-figli
- **Introdurre una routine di attività fisica:** selezionare giorni settimanali dedicati al movimento (es. passeggiate serali, uscite nel fine settimana)
- **Limitare il tempo sedentario davanti agli schermi**: regolare l'uso di dispositivi digitali e proporre alternative stimolanti che favoriscano l'interazione e la creatività
- **Condivisione**: coinvolgere l'intera famiglia in attività motorie e promuovere comportamenti salutari, per un modello positivo e attivo
- **Incoraggiare lo sport e le attività di gruppo**: sostenere la partecipazione ad attività sportive organizzate o laboratori ludico-motori per costruire e potenziare competenze motorie e relazionali

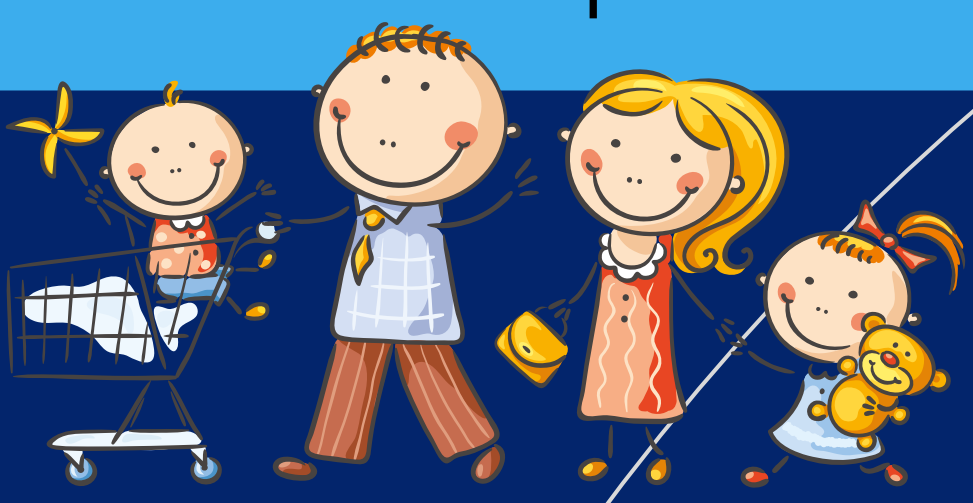

ATTIVITÀ SPORTIVA E SANA ALIMENTAZIONE

La strategia alimentare più efficace consiste nell'adottare un **regime nutrizionale equilibrato e diversificato**, in grado di garantire un apporto completo e bilanciato di tutti i macronutrienti e micronutrienti essenziali. Un'adeguata alimentazione rappresenta, infatti, un fattore determinante per l'**ottimizzazione della performance fisica** e per il **mantenimento del benessere psicofisico**

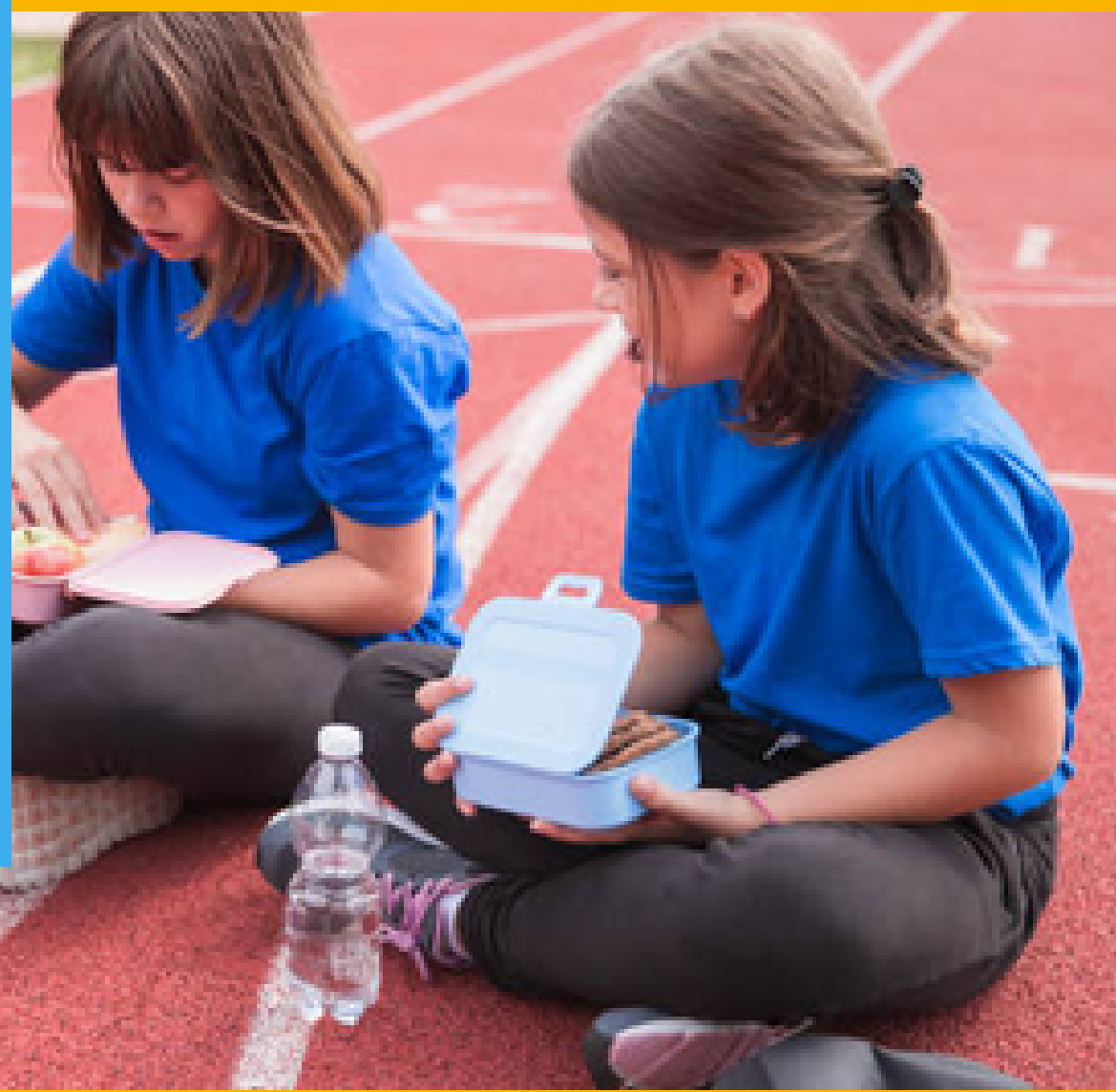

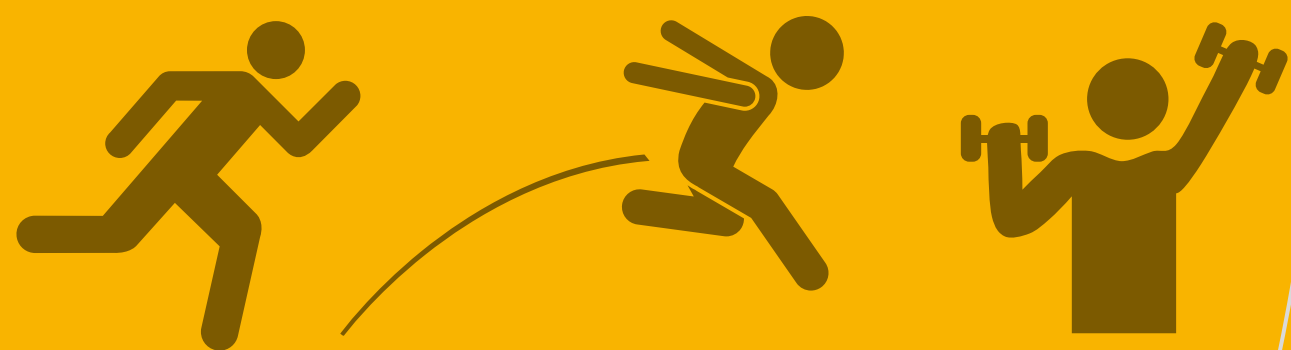

UN ESEMPIO DI DIETA EQUILIBRATA

Prima colazione: Latte, biscotti, yogurt con l'aggiunta di qualche fettina di prosciutto crudo magro e di formaggio fresco.

Spuntino di metà mattinata: Panino con prosciutto, frutta, dolce da forno

Pranzo: Una piccola porzione di pasta o riso condita con sugo di pomodoro, olio d'oliva e un po' di parmigiano

Cena: Costituita da un pasto completo con presenza di verdure e frutta fresca. Il classico minestrone all'italiana è un' ottima soluzione per la cena degli sportivi. A seguire una giusta porzione di carne o di pesce.

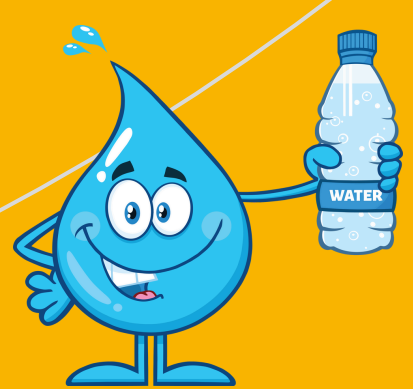

*È fondamentale **non saltare mai la colazione:** rappresenta il pasto che riattiva il metabolismo dopo il digiuno notturno e fornisce l'energia indispensabile per iniziare la giornata in modo attivo e concentrato*

*L'**idratazione** è un elemento imprescindibile per il corretto funzionamento dell'organismo, specialmente in presenza di attività fisica. È essenziale bere regolarmente nel corso della giornata, prima ancora di percepire la sete*

CONCLUSIONI

Per contrastare la **sedentarietà** è essenziale promuovere l'**esercizio fisico adattato**, come strumento di inclusione e partecipazione attiva in sinergia con un'alimentazione sana. In riferimento al contesto scolastico questi elementi hanno l'obiettivo di favorire la **crescita consapevole di ogni studente**

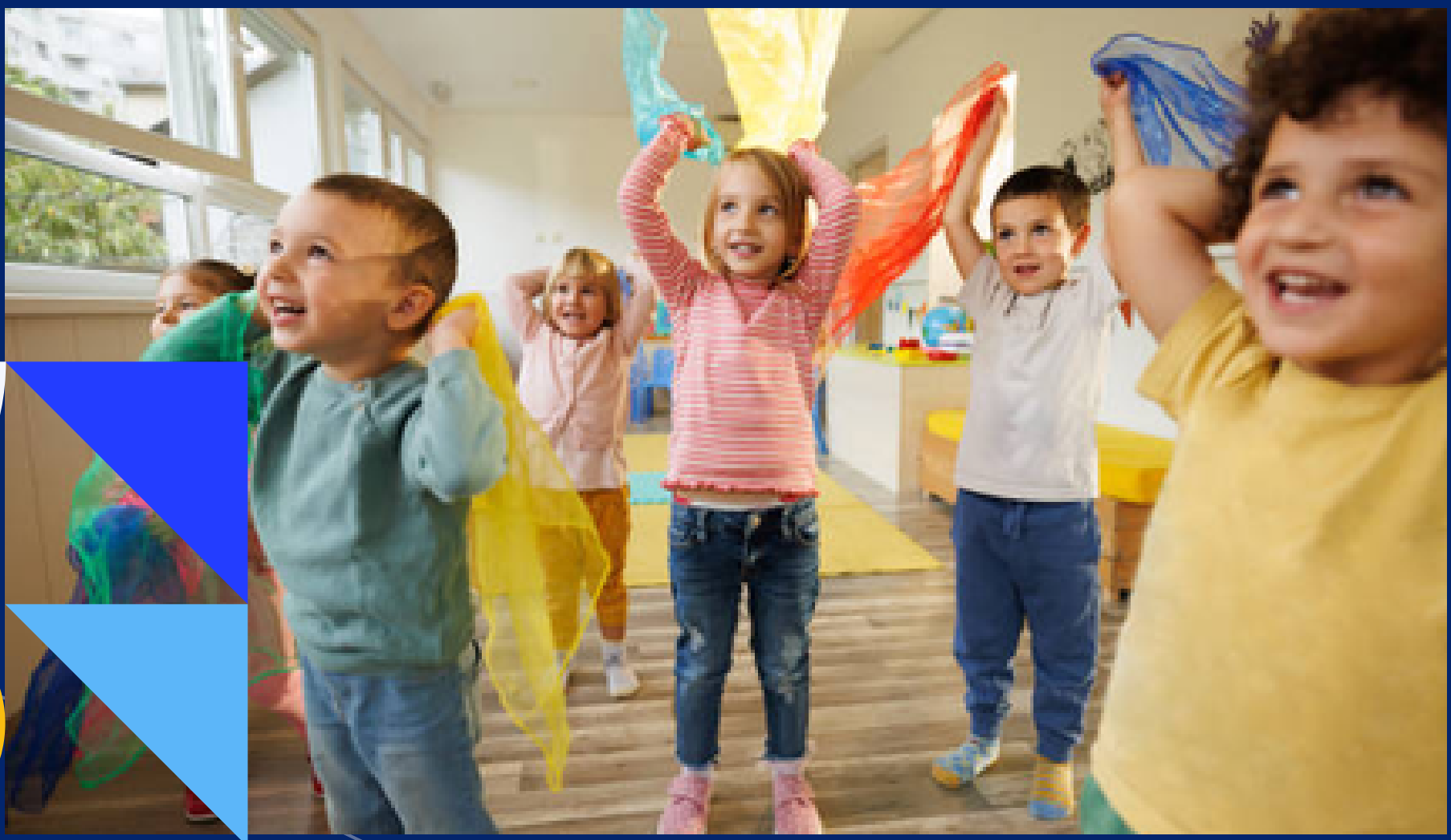

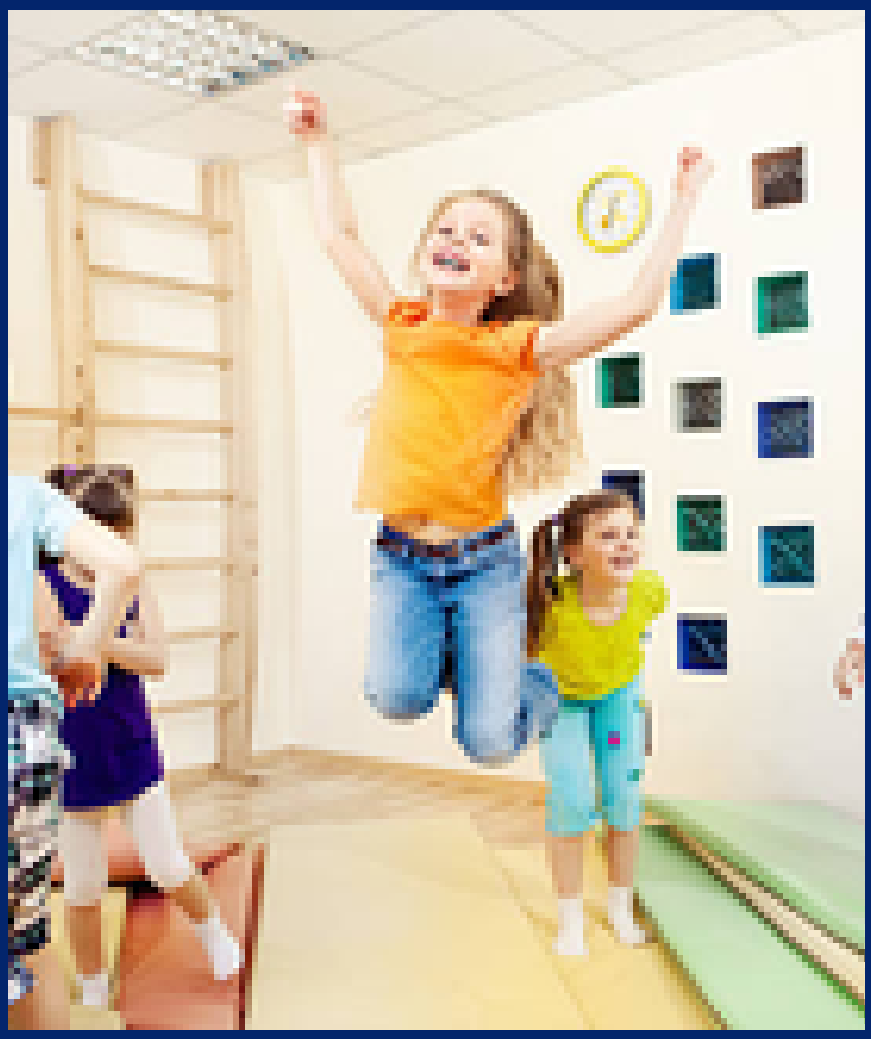

TAKE HOME MESSAGE

- **Piacevolezza e aderenza** all'esercizio fisico: proporre esperienze motorie gratificanti e accessibili a tutti riduce la sedentarietà e potenzia la motivazione, facilitando l'adozione di comportamenti salutari e sostenibili nel tempo
- **Stimolazione delle funzioni esecutive e delle capacità attentive:** l'esercizio fisico contribuisce a contrastare la sedentarietà migliorando le funzioni esecutive, l'attenzione selettiva e la memoria di lavoro
- **Promozione delle dinamiche relazionali:** il movimento offre occasioni per superare l'isolamento legato alla sedentarietà, favorendo le interazioni sociali, la comunicazione e la costruzione di legami positivi

Promuovere la pratica di attività fisica quotidiana significa sostenere uno sviluppo armonico, favorire il benessere psicofisico e consolidare abitudini salutari nel tempo. Citando Giovenale "**Mens sana in corpore sano**" si può racchiudere il nostro pensiero e ricordiamo che **piccole azioni quotidiane possono generare grandi cambiamenti**

"La sedentarietà spegne l'energia, il movimento la riaccende"

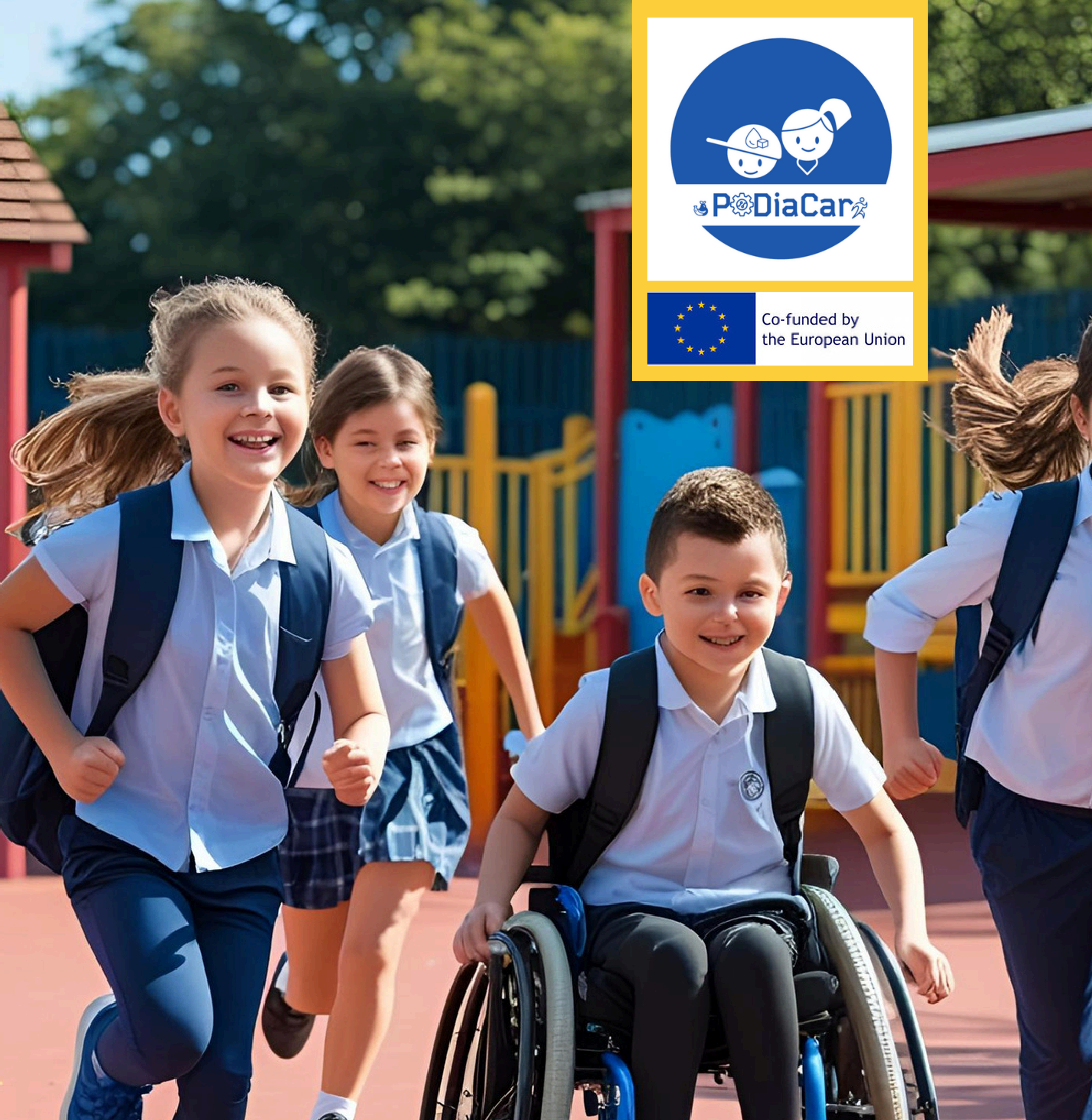

PODiaCar Team

Ospedale dei Bambini "Vittore Buzzi" e Università di Milano (Italia): Gianvincenzo Zuccotti, Valeria Calcaterra, Elvira Verduci, Savina Mannarino, Virginia Rossi, Martina Tosi, Giulia Fiore

Università degli Studi di Pavia (Italia): Matteo Vandoni, Luca Marin, Alessandro Gatti, Caterina Cavallo, Vittoria Carnevale Pellino, Agnese Pirazzi

Università di Granada (Spagna): Cristina Campoy, Mireia Escudero Marin, Rocio Bonillo Leon, Mercedes García-Bermúdez, Antonio E. Jerez-Calero, Inmaculada Guerrero, Ricardo Pérez Iañez, José Mª Gómez Vida

Asomi College of Malta (Malta): Valter Pagani, Umberto Ciriello, Francesco Conti

LUNEX University (Lussemburgo): Camilo Corbellini, Khatija Bhadur
